# Silencing of CD147 inhibits cell proliferation, migration, invasion, lipid metabolism dysregulation and promotes apoptosis in lung adenocarcinoma via blocking the Rap1 signaling pathway

**DOI:** 10.1186/s12931-023-02532-0

**Published:** 2023-10-25

**Authors:** Ning Zhang, Zhouzhong Liu, Xuwang Lai, Shubin Liu, Yuli Wang

**Affiliations:** 1 Department of Gastroenterology, Ganzhou People’s Hospital, the Affiliated Ganzhou Hospital of Nanchang University, Ganzhou City, 341000 Jiangxi Province China; 2 Department of Oncology, Ganzhou People’s Hospital, the Affiliated Ganzhou Hospital of Nanchang University, Ganzhou City, 341000 Jiangxi Province China

**Keywords:** Lung adenocarcinoma, CD147, Transcriptome sequencing, Lipid metabolism, Rap1 signaling pathway

## Abstract

**Objective:**

CD147 is an important glycoprotein that participates in the progression of diverse cancers. This study aims to explore the specific function of CD147 in lung adenocarcinoma (LUAD) and to reveal related downstream molecular mechanisms.

**Methods:**

Followed by silencing of CD147, the viability, migration, invasion, and apoptosis of LUAD cells were measured by CCK8, wound healing, transwell assay, and flow cytometer, respectively. The expression of CD147 and two markers of lipid metabolism (FASN and ACOX1) were detected by qRT-PCR. A xenograft tumor model was constructed to investigate the function of CD147 *in vivo.* Then transcriptome sequencing was performed to explore the potential mechanisms. After measuring the expression of Rap1 and p-p38 MAPK/p38 MAPK by western blot, the changes of CD147 and lipid metabolism markers (FASN, ACOX1) was detected by Immunohistochemistry. Moreover, a Rap1 activator and a Rap1 inhibitor were applied for feedback functional experiments.

**Results:**

CD147 was up-regulated in LUAD cells, and its silencing inhibited cell proliferation, migration, invasion, lipid metabolism dysregulation and promoted apoptosis, while overexpression of CD147 showed the opposite results. Silencing of CD147 also inhibited the growth of tumor xenografts in mice. Transcriptome sequencing revealed 834 up-regulated differentially expressed genes (DEGs) and 602 down-regulated DEGs. After functional enrichment, the Rap1 signaling pathway was selected as a potential target, which was then verified to be blocked by CD147 silencing. In addition, the treatment of Rap1 activator weakened the inhibiting effects of si-CD147 on the proliferation, migration, invasion, and lipid metabolism in LUAD cells, while the intervention of RAP1 inhibitor showed the opposite results.

**Conclusions:**

Silencing of CD147 inhibited the proliferation, migration, invasion, lipid metabolism dysregulation and promoted apoptosis of LUAD cells through blocking the Rap1 signaling pathway.

**Supplementary Information:**

The online version contains supplementary material available at 10.1186/s12931-023-02532-0.

## Introduction

Non-small cell lung cancer (NSCLC) is a common form of lung cancer, making up to approximately 85% proportion [1]. Adenocarcinoma, squamous cell carcinoma (SCC) and large cell carcinoma all belong to NSCLC [2]. Among them, lung adenocarcinoma (LUAD) is the most common histological type and the main cause of cancer death worldwide [3]. The main treatment options for LUAD include surgical resection, chemotherapy, radiotherapy, and molecular targeted therapy, but the prognosis usually performs poorly. Therefore, it is necessary to explore novel targets for LUAD treatment.

CD147, also known as extracellular matrix metalloproteinase inducer, is a kind of glycoprotein that is involved in the regulation of extracellular matrix remodeling [4]. Via regulating glycolysis, matrix degradation, metastasis, and angiogenesis, CD147 exerts a critical role in the diverse types of cancers, which is considered a diagnostic and therapeutic target [5]. Previous researches have determined the diagnostic and prognostic potential of CD147 in lung cancer. CD147 accelerates osteoclast formation induced by lung cancer via regulating IL-8 secretion [6]. Up-regulation of CD147 in NSCLC is positively related to TNM stage, lymph node metastasis, and poor overall survival [7], and CD147 contributes to a poor response to cisplatin treatment and a poor prognosis in patients with advanced NSCLC [8]. Similarly, CD147 and MMP-2 can be used as an objective marker for predicting SCC and LUAD [9]. In addition, CD147 enhances glucose metabolism in LUAD, which may be a promising therapeutic target for LUAD [10]. However, researches on the action mechanisms of CD147 in LUAD is rare.

As a conserved telomere-interacting protein, Rap1 acts a key regulator in integrin- and cadherin-related processes [11]. Rap1 is closely involved in oncogenesis, progression, and chemoresistance in human cancers, acting as a tumor promoter [12]. Evidence has also determined that the activated Rap1 signaling pathway can promote tumor progression. For example, Mex3a enhances cell proliferation and invasion through activating the RAP1/MAPK signaling pathway in colorectal cancer [13]. GREM1 accelerates the migration, invasion, and epithelial-mesenchymal transition of NSCLC cells via activating the Rap1 signaling pathway [14]. However, it is still unknown whether CD147 regulates Rap1 signaling pathway in LUAD.

The dysregulation of lipid metabolism is a prominent metabolic abnormality in cancer [15]. Because the enhancement of lipid uptake, storage, and lipogenesis contributes to tumor growth, lipid metabolism reprogramming is considered as a potential therapeutic target for cancer [16]. A previous study has determined that CD147 reprograms fatty acid metabolism via regulating Akt/mTOR/SREBP1c and P38/PPARα pathways, which is related to the proliferation and metastasis of hepatocellular carcinoma cells [17]. In this research, the regulatory role of CD147 on lipid metabolism was also analyzed in LUAD. The Rap1 signaling pathway identified by transcriptome sequencing was selected as a research target, which may be involved in the action mechanisms of CD147 in LUAD. Our study was designed to reveal the specific function and related mechanisms of CD147 in LUAD, providing potential therapeutic targets.

## Materials and methods

### Cell culture and treatments

Two LUAD cell lines (A549 and H1299) and a normal human lung epithelial cell line(BEAS-2B) were purchased from ATCC (Manassas, VA, USA). LUAD cells were cultured in DMEM containing 10% fetal bovine serum (FBS), and BEAS-2B cells were cultured in Epithelial Cell Culture Medium containing 10% FBS. Two siRNAs targeting CD147(si-CD147-1 and si-CD147-2) and corresponding negative control (si-NC) were synthesized from Ribobio (Guangzhou, China). The overexpression vector of CD147 (ov-CD147) and corresponding negative control (ov-NC) were purchased from Ribobio. The sequences of siRNAs were shown in Table 1. LUAD cells were transfected with siRNAs and the overexpression vectors for 48 h using Lipofectamine 3000 (Thermo Fisher Scientific, Waltham, MA, USA). si-CD147-transfected A549 cells also received the treatment of a Rap1 activator (8-pCPT-2’-O-Me-cAMP; Univ, Shanghai, China) and a Rap1 inhibitor (GGTI298, Univ) for another 30 min.

**Table 1:** The sequences of siRNAs and primers

siRNAs/primers	Sequences (5’-3’)
si-CD147-1-sense	GGUCAGAGCUACACAUUGATT
si-CD147-1-antisense	UCAAUGUGUAGCUCUGACCTT
si-CD147-2-sense	GUGAAGUCGUCAGAACACATT
si-CD147-2-antisense	UGUGUUCUGACGACUUCACTT
si-NC-sense	UUCUCCGAACGUGUCACGUTT
si-NC-antisense	ACGUGACACGUUCGGAGAATT
CD147-F	GACGACCAGTGGGGAGAGTA
CD147-R	GGCCTTGTCCTCAGAGTCAG
FASN-F	CTGGCTCAGCACCTCTATCC
FASN-R	CAGGTTGTCCCTGTGATCCT
ACOX1-F	CTGAAGGCTTTCACCTCCTG
ACOX1-R	CATGCCACACACCAACTTTC
GAPDH-F	CCTTCCGTGTCCCCACT
GAPDH-R	GCCTGCTTCACCACCTTC

### Quantitative real-time PCR (qRT-PCR)

Total RNAs were extracted from cells by lysing in TRIzol reagent (Thermo Fisher Scientific). After quantified by a microplate reader (Multiskan SkyHigh, Thermo Fisher Scientific) referring to the optical density (OD) at 260/280 nm, the RNAs were reversely transcribed into cDNAs using a cDNA Reverse Transcription Kit (Applied Biosystems, Foster City, CA, USA). qRT-PCR was run on ABI7500 (Applied Biosystems) using the following program of 95°C for 10 min, and 36 cycles of 95°C for 10 s and 60°C for 30 s. The expression level was calculated by the 2^−∆∆Ct^ method. GAPDH was used as an internal control. The primers were synthesized from TaKaRa (Dalian, China) and the sequences were shown in Table 1.

### Cell counting Kit-8 (CCK8) assay

The viability of LUAD cells was detected using a CCK8 kit (Solarbio, Beijing, China) following the instructions. Briefly, LUAD cells that seeded in 96-well plates at a density of 1 × 10^4^/well were cultured for 0, 24, 48, 72, and 96 h, respectively. Cells that collected at different time points were then treated with CCK8 reagent for 2 h. The OD at 450 nm was finally measured under a microplate reader (Thermo Fisher Scientific).

### Wound healing assay

The migration of LUAD cells was measured by wound healing assay. Briefly, LUAD cells that seeded in 6-well plates were cultured until complete attachment. A wound gap was then made on the plate using a pipette tip. Followed by removing the cell debris, cells were subsequently cultured for 24 h. The wound distance that measured at 0 and 24 h post-wounding was used for evaluating cell migration.

### Trans-well assay

The invasion of LUAD cells was measured using trans-well assay (Corning, Kenneburg, ME, USA). Briefly, the upper chamber was per-coated with matrigel and then filled with LUAD cells at a density of 2 × 10^5^/chamber. The lower chamber was filled with DMEM containing 15% FBS. At 24 h post-culturing, cells in the lower chamber were fixed in 4% paraformaldehyde and stained with 0.5% crystal violet. Finally, invasive cells with violet staining were counted under a microscope (Olympus, Japan).

### Apoptosis test

According to the instructions of manufacturer, LUAD cells were collected and incubated with Annexin V-FITC and propidium iodide (Solarbio) for 15 min. The fluorescence intensity of the cells was detected by flow cytometry and the apoptosis rate of LUAD cells was evaluated.

### Lentivirus infection

Lentivirus harboring LV-siCD147-1 (LV-CD147) and LV-siNC (LV-NC) were generated by GeneChem Co., Ltd (Shanghai, China). A549 cells (2 × 10^6^) were seeded in 10 cm dish, and then infected with lentivirus carrying the siRNA or NC at MOI value of 10. After 72 h of 1 µg/mL puromycin selection, A549 cells were collected for qRT-PCR analysis.

### Establishment of xenograft tumor model in mice

Total 12 BALB/c nude mice at 6 weeks old (Shanghai Institute of Material Medical, Shanghai, China) were used for model establishment. Briefly, A549 cells stably transfected with LV-CD147 or LV-NC were injected subcutaneously into the right flank of mice (N = 6 each group) to conduct a LUAD model as previous reported [18]. The tumor volume was measured every week. After the last measurement at the 4th week, mice were anesthetized via intraperitoneal injection of pentobarbital sodium (50 mg/kg) and immediately sacrificed by cervical dislocation. The removed tumor xenografts were weighed and collected for subsequent experiments. The animal experiments were approved by the local ethical committee following the Guide for the Care and Use of Laboratory Animals.

### Immunohistochemistry (IHC) analysis

The tissue was fixed with 4% paraformaldehyde, embedded in paraffin and sectioned. The paraffin-embedded sections were dewaxed and rehydrated, and EDTA was used for antigen repair. At room temperature, sections were incubated with 3% H_2_O_2_ to quench endogenous peroxidase activity. Afterwards, samples were blocked with BSA and incubated overnight at 4℃ with specific primary antibodies, including anti-CD147 (ab212057, 1:200, Abcam, Cambridge, USA), anti-ACOX1 (ab184032, 1:200, Abcam), anti-FASN (ab128856, 1:200, Abcam). Later, samples were incubated for 1 h at 37°C with HRP-labeled goat anti-Rabbit secondary antibodies. The samples were then dyed with DAB and hematoxylin before sealing.

### Western blot

The protein samples were extracted from LUAD cells or tumor xenografts via lysing in RIPA buffer (Solarbio). After quantification, equal protein samples were separated by 10% SDS-PAGE, and transferred onto PDVF membrane (Millipore, Danvers, USA). After blocked with skim milk for 2 h, the membranes received another 12 h of incubation with primary antibodies, including anti-CD147, ACOX1, FASN, Rap1, p38 MAPK, p-p38 MAPK, and GAPDH (Abcam). After 2 h of incubation with HRP-conjugated IgG (Abcam), the blots in the membrane were visualized using an ECL kit (Thermo Fisher Scientific) and finally quantified using an Imaging Analysis System (Tanon5200, Shanghai, China).

### Transcriptome sequencing analysis

Total RNAs isolated from A549 cells in the si-CD147 or si-NC groups were collected for transcriptome sequencing. Briefly, mRNAs were separated from total RNAs by polyA-selection and fragmented into fragments at 200–300 bp. The cDNA library (about 450 bp) constructed by mRNAs was then sequenced on Illumina Novaseq 6000 platform. The differentially expressed genes (DEGs) were identified according to |log_2_FoldChange| > 1 and P < 0.05, and clustered by Pheatmap. Gene ontology (GO) analysis and Kyoto Encyclopedia of Genes and Genomes (KEGG) pathway analysis were performed for functional enrichment of the isolated DEGs. In addition, a protein-protein interaction (PPI) network was established using STRING with a confidence score of 0.15, which was visualized using Cytoscape.

### Statistical analysis

The data generated in this study were analyzed using the software of Prism 8.0 (GraphPad, San Diego, CA, USA), presenting mean ± standard deviation. The comparisons between two groups were determined by t test, and those among multiple groups were determined by one-way ANOVA and subsequent Tukey’s test. P value less than 0.05 represented statistically significant.

## Results

### Silencing of CD147 inhibits the proliferation, migration, invasion, lipid metabolism and promotes apoptosis of LUAD cells

**Fig. 1:**
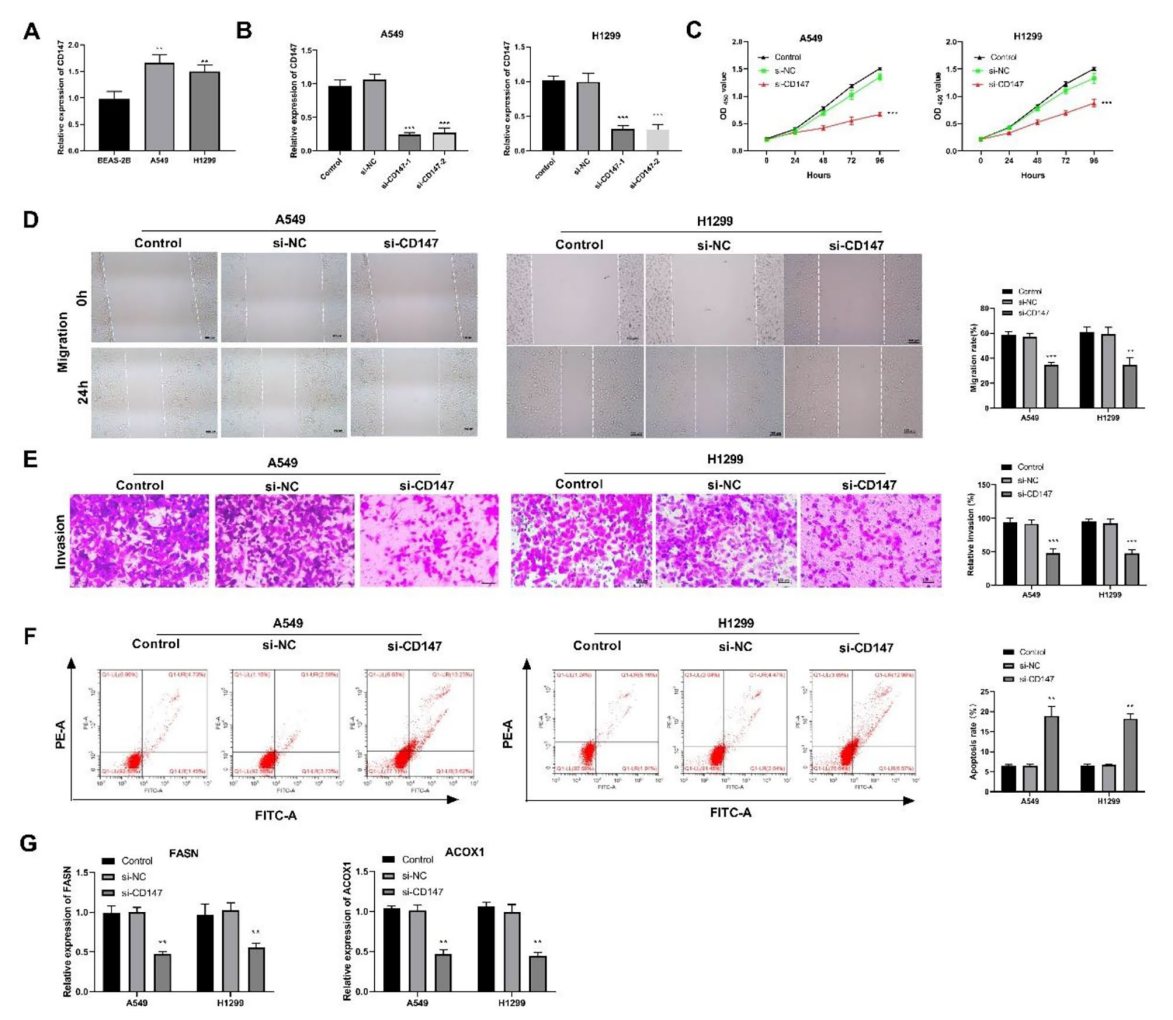
Silencing of CD147 inhibited the proliferation, migration, invasion, and lipid metabolism of LUAD cells. **(A)** The mRNA expression of CD147 in two LUAD cell lines (A549 and H1299 cells) and a normal human lung epithelial cell line (BEAS-2B cells) was detected by qRT-PCR; **p < 0.01 vs. BEAS-B cells. **(B)** The silencing efficiency of si-CD147-1/2 in LUAD cells was detected by qRT-PCR. C-F. The viability, migration, invasion, and apoptosis of transfected LUAD cells was detected by CCK8 assay, wound healing assay, transwell assay, and flow cytometry, respectively. G. The protein expression of FASN and ACOX1, two key genes involved in lipid metabolism was detected by western blot. ^*^p < 0.05, ^**^p < 0.01, ^***^p < 0.001 vs. Control

The mRNA expression of CD147 was detected in LUAD cells by qRT-PCR. There was a significantly higher mRNA expression of CD147 in A549/H1299 cells than that in BEAS-2B cells (P < 0.01; Fig. 1A). To evaluate the function of CD147 in LUAD, CD147 was silenced in A549 and H1299 cells by the transfection of si-CD147-1/2 (P < 0.001). si-CD147-1 with a relatively high silencing efficiency was used for following assays (si-CD147) (Fig. 1B). Functional assays determined that the transfection of si-CD147 significantly decreased the viability, migration, and invasion of A549/H1299 cells, and promoted apoptosis rate (P < 0.001; Fig. 1C-F). In addition, silencing CD147 also decreased the mRNA expression of two key genes FASN and ACOX1, which are involved in lipid metabolism (P < 0.01; Fig. 1G).

### Overexpression of CD147 promotes the proliferation, migration, invasion, lipid metabolism and inhibits apoptosis of LUAD cells

**Fig. 2:**
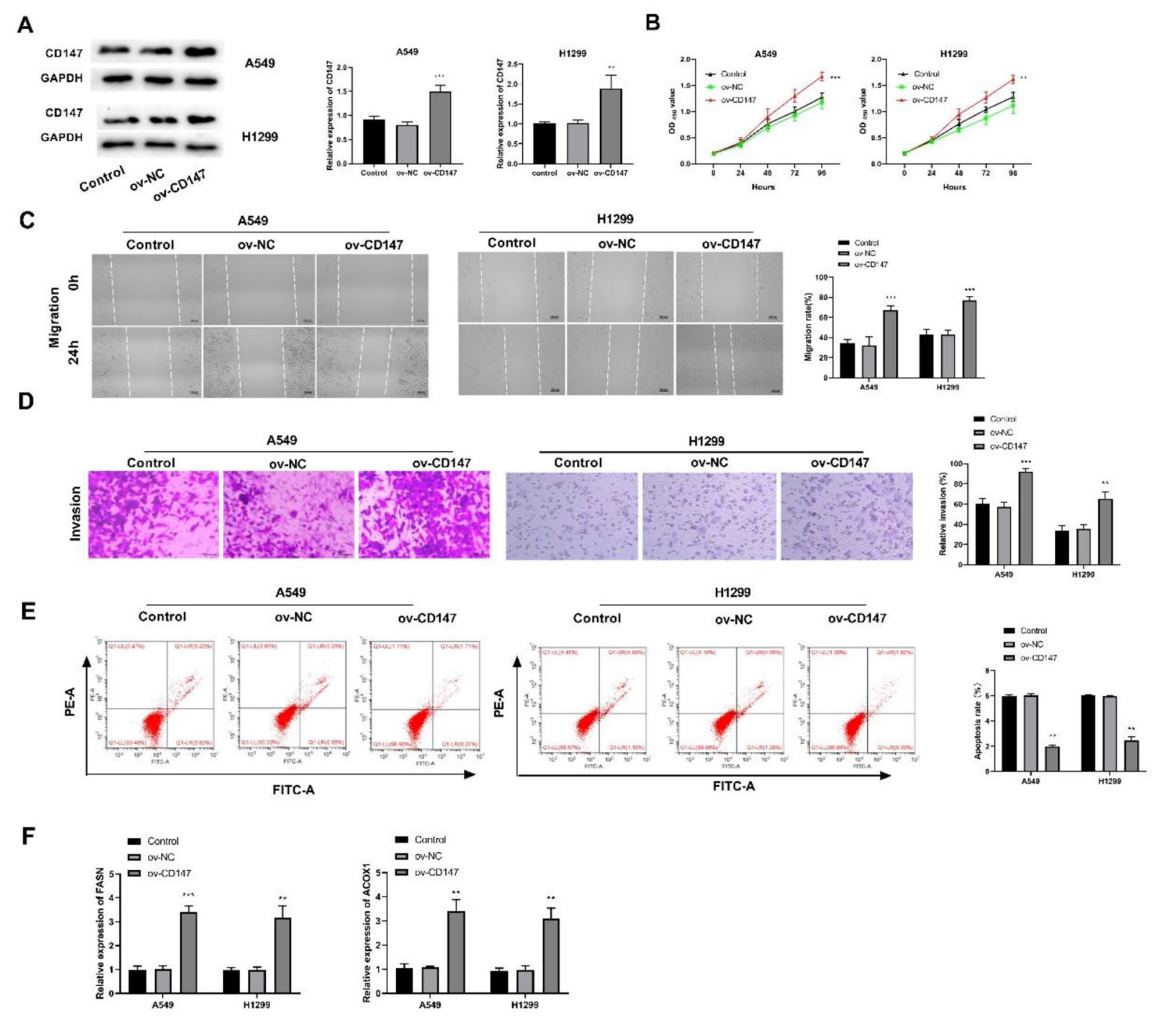
Overexpression of CD147 promoted the proliferation, migration, invasion, lipid metabolism of LUAD cells. A. The protein and mRNA expression of CD147 in A549 and H1299 cells was detected by western blot and qRT-PCR; **p < 0.01 vs. control. B-E. The viability, migration, invasion, and apoptosis of transfected LUAD cells was detected by CCK8 assay, wound healing assay, transwell assay, and flow cytometry, respectively. F. The protein expression of FASN and ACOX1, two key genes involved in lipid metabolism was detected by western blot. ^*^p < 0.05, ^**^p < 0.01, ^***^p < 0.001 vs. Control

In order to further explore the function of CD147 in LUAD, CD147 was over expressed in A549 and H1299 cells by the transfection of ov-CD147 (P < 0.001). The overexpression efficiency of CD147 in LUAD cells was detected by western blot (Fig. 2A). The result of cell function assays showed that the transfection of ov-CD147 significantly increased the viability, migration, and invasion of A549/H1299 cells, while inhibited apoptosis compared to the control group (P < 0.001; Fig. 2C-E). Besides, overexpression of CD147 increased the mRNA expression of FASN and ACOX1 (P < 0.01; Fig. 2F).

### Silencing of CD147 represses the growth of tumor xenografts in mice

**Fig. 3:**
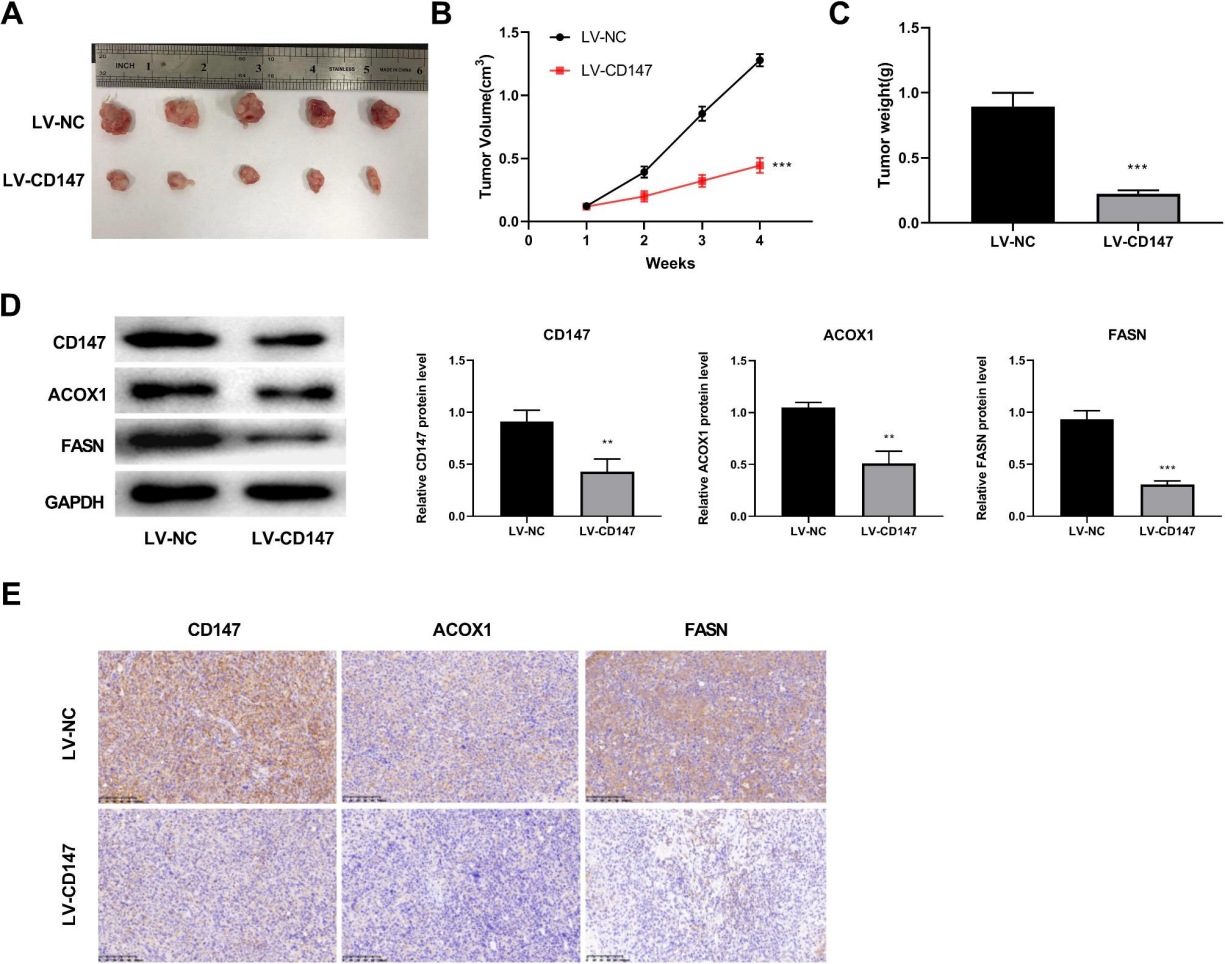
Silencing of CD147 inhibits the growth of tumor xenografts in mice. **A.** The morphology of tumor xenografts. **B-C**. The volume and weight of tumor xenografts. **D.** The protein expression of CD147, FASN, and ACOX1 in tumor xenografts was detected by western blot. **E.** The changes of CD147 and lipid metabolism markers (FASN, ACOX1) was detected by immunohistochemistry (Amplification: 200×, Scale: 100 μm). ^**^p < 0.01, ^***^p < 0.001 vs. LV-NC

A tumor xenograft model was constructed to determine the anti-tumor potential of silencing CD147 in vivo. As shown in Fig. 3A-C, mice treated with LV-CD147 presented significantly lower tumor volume and weight than mice treated with LV-NC (P < 0.001; Fig. 3A-C). The down-regulation of CD147 in tumor tissues was confirmed by western blot following injection of LV-CD147 (P < 0.01). In addition, silencing of CD147 also down-regulated FASN and ACOX1 in tumor tissues (P < 0.01; Fig. 3D-E).

### Screening and functional enrichment of DEGs in CD147-silenced cells

**Fig. 4:**
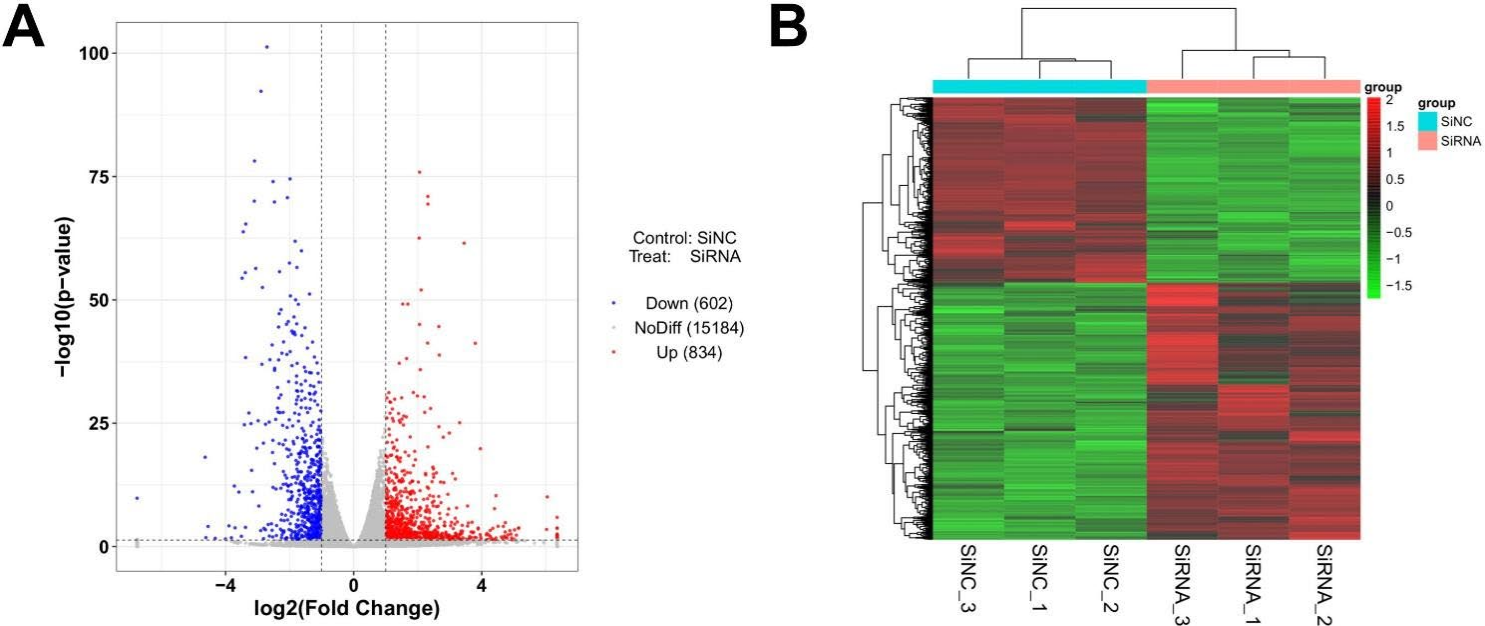
The isolation of differentially expressed genes (DEGs) in CD147-silenced cells. **(A)** A volcano map of DEGs; red points presented up-regulated DEGs, and blue points presented down-regulated DEGs. **(B)** A heatmap of DEGs; red blocks presented up-regulated DEGs, and green blocks presented down-regulated DEGs

To reveal the underlying action mechanisms of CD147 at the molecular level, transcriptome sequencing was performed in CD147-silenced cells. Total 1436 DEGs were determined, including 834 up-regulated and 602 down-regulated DEGs (Fig. 4A and B). The detailed information of the top 10 up-regulated/down-regulated DEGs were listed in Table 2.

**Table 2:** Top 10 up-regulated and 10 down-regulated DEGs in CD147-silenced LUAD tissues

Name	Description	log_2_ Fold Change	P-value	up/down
C6orf58	chromosome 6 open reading frame 58	6.044861121	8.65716E-11	up
PROX1	prospero homeobox 1	6.0239283	0.000337854	up
TTC24	tetratricopeptide repeat domain 24	5.156042601	0.000173806	up
SH2D1B	SH2 domain containing 1B	5.148474667	0.000200026	up
NPC1L1	NPC1 like intracellular cholesterol transporter 1	5.081381764	0.004983105	up
OPRD1	opioid receptor delta 1	5.023649494	0.000421111	up
PHF24	PHD finger protein 24	5.018362347	0.033310134	up
DNAH12	dynein axonemal heavy chain 12	4.952275926	0.045419643	up
ODF3	outer dense fiber of sperm tails 3	4.934087943	0.018475317	up
DAPP1	dual adaptor of phosphotyrosine and 3- phosphoinositides 1	4.912562984	0.003041524	up
KRT4	keratin 4	-6.763037823	1.62743E-10	down
RIBC2	RIB43A domain with coiled-coils 2	-4.638075823	7.90393E-19	down
ANKRD30A	ankyrin repeat domain 30 A	-4.613759903	0.015543631	down
LYPD6	LY6/PLAUR domain containing 6	-4.546836521	8.62627E-05	down
AHSG	alpha 2-HS glycoprotein	-4.323765937	0.02223893	down
KIF5C	kinesin family member 5 C	-4.001462641	0.028973629	down
PSAPL1	prosaposin like 1	-3.894673678	6.44995E-05	down
S1PR5	sphingosine-1-phosphate receptor 5	-3.818536614	0.016695668	down
FAM111B	FAM111 trypsin like peptidase B	-3.725329602	5.34734E-13	down
PTK7	protein tyrosine kinase 7 (inactive)	-3.703773544	0.000162958	down

**Fig. 5:**
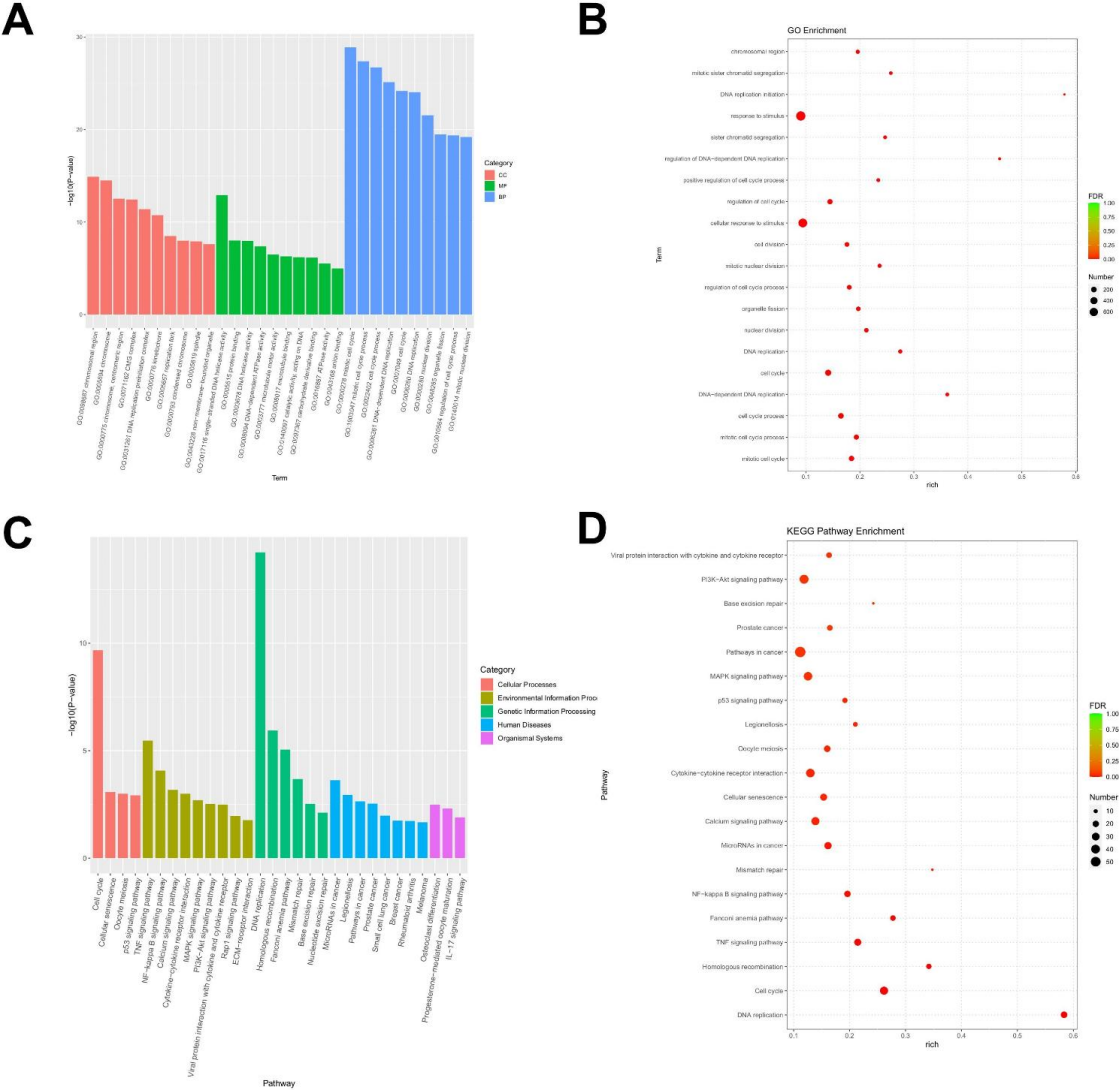
The functional enrichment of DEGs isolated from CD147-silenced tissues. **(A)** A bar plot of GO enrichment. **(B)** A bubble plot of GO enrichment. **(C)** A bar plot of KEGG enrichment. **(D)** A bubble plot of KEGG enrichment

The DEGs were subsequently annotated and classified to reveal their potential functions. Based on GO enrichment analysis, the DEGs were mainly enriched in chromosomal region, chromosome, CMG complex (cellular components, CC), single-stranded DNA helicase activity, protein binding, DNA helicase activity (molecular function, MF), cell cycle, DNA-dependent DNA replication (biological process, BP), etc. (Fig. 5A). The top 20 GO terms referring to the FDR value were listed in Fig. 4B. On the other hand, KEGG enrichment analysis revealed many signaling pathways were enriched, mainly including p53, TNF, NF-kB, Calcium, MAPK, PI3K-Akt, and Rap1 signaling pathways (Fig. 5C). Referring to the FDR values, the top 20 KEGG terms were listed in Fig. 5D.

**Fig. 6:**
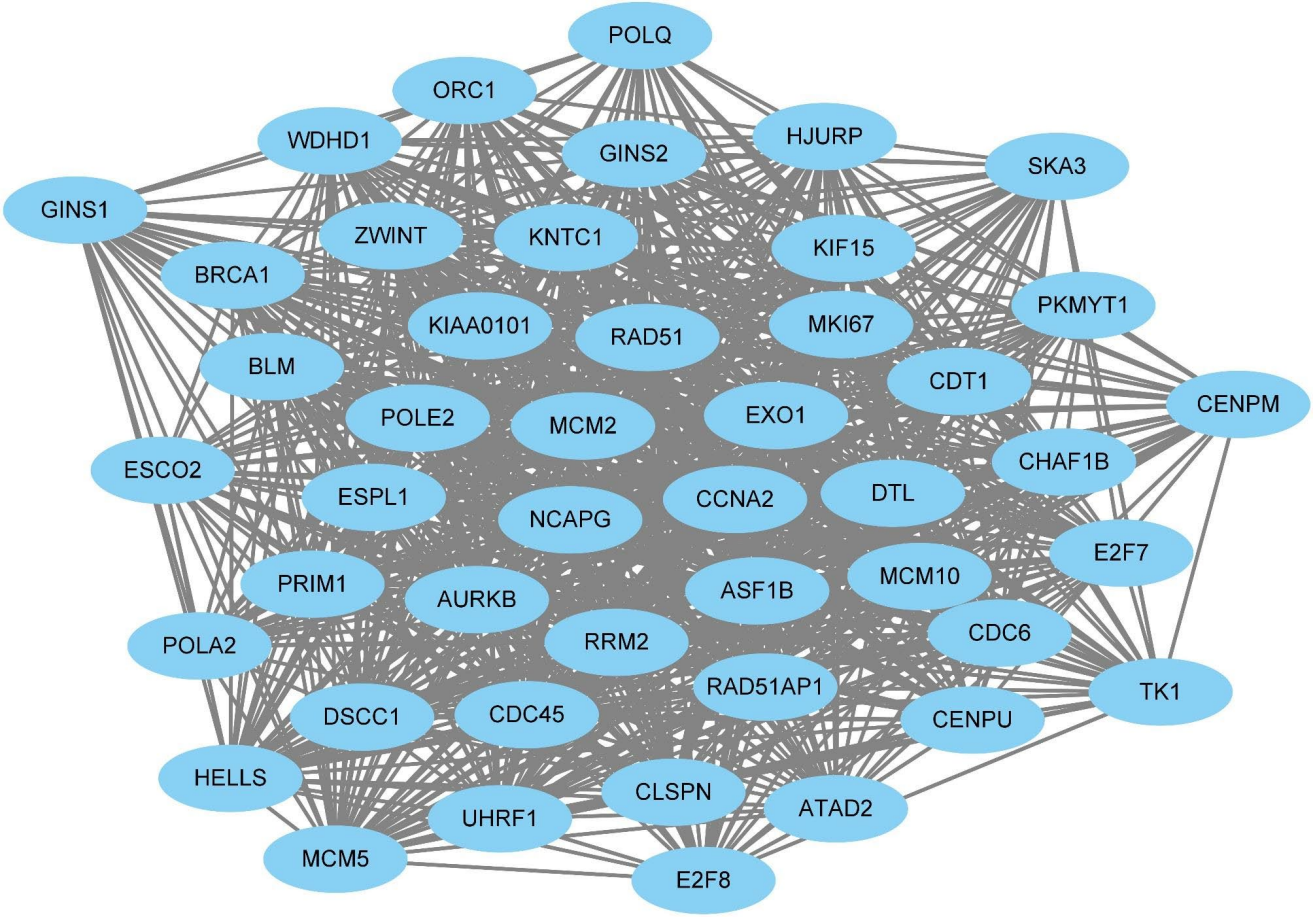
A PPI network consists of 46 hub genes; Lines between nodes represented the presence of interactions

Furthermore, a PPI network, including 46 hub genes was established (Fig. 6). Some of these hub genes exerted critical regulatory roles in the progression of LUAD, such as MCM2, CCNA2, KNTC1, POLE2, and KIAA0101.

### Silencing of CD147 blocks the Rap1 signaling pathway in LUAD

**Fig. 7:**
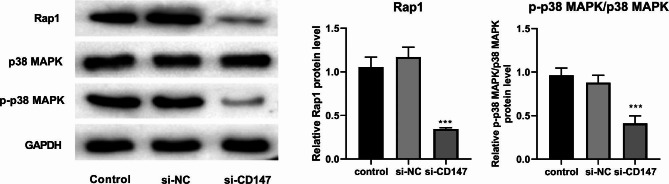
Silencing of CD147 blocks the Rap1 signaling pathway. The protein expression of Rap1 and p-p38 MAPK/38 MAPK was detected by western blot. ^**^p < 0.01, ^***^p < 0.001 vs. Control

As described above, the Rap1 signaling pathway was a potential downstream pathway of CD147 that enriched by DEGs. Since Rap1 signaling pathway participated in the progression of LUAD, whether CD147 could regulate Rap1 signaling pathway was verified. Then, we found that the protein expression of Rap1 and p-p38 MAPK/p38 MAPK was decreased by the intervention of si-CD147 (P < 0.01; Fig. 7), but not by the intervention of si-NC. These results indicated that the Rap1 signaling pathway was blocked by CD147 silencing in LUAD.

### The effect of Rap1 signaling pathway on the regulation of CD147

**Fig. 8:**
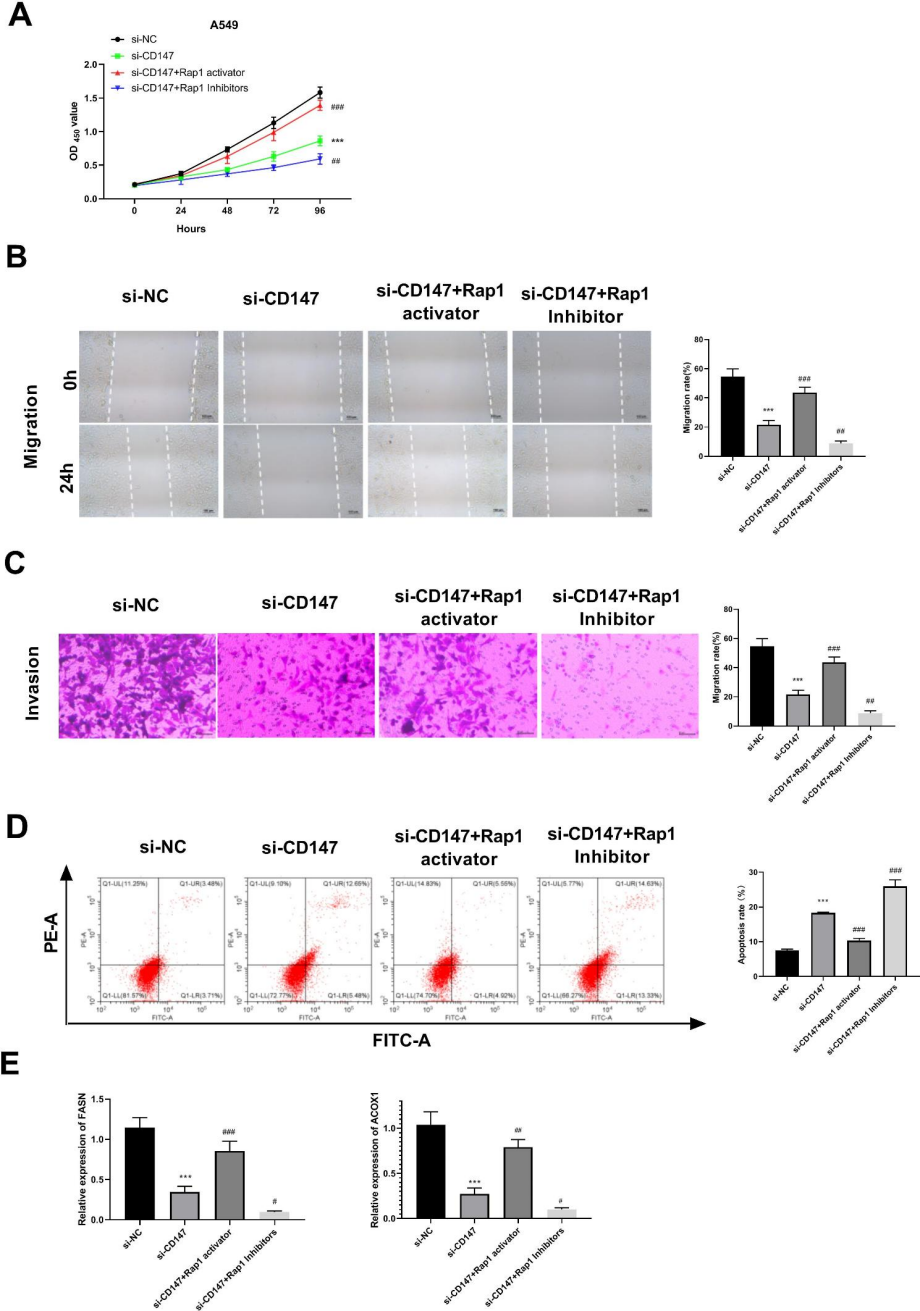
The effects of Rap1 activator on the proliferation, migration, invasion, and lipid metabolism of CD147-silenced A549 cells. **A-D.** The viability, migration, invasion, and apoptosis of treated A549 cells was detected by CCK8 assay., wound healing assay, transwell assay, and flow cytometry, respectively. E. The protein expression of FASN and ACOX1, two key genes involved in lipid metabolism was detected by western blot. ^***^p < 0.001 vs. si-NC; ^##^p < 0.01, ^###^p < 0.001 vs. si-CD147

To further confirm whether the regulatory mechanism of CD147 in LUAD was related to the Rap1 signaling pathway, a Rap1 activator and a Rap1 inhibitor were used to active and inhibit the Rap1 signaling pathway, respectively. The following functional assays showed that Rap1 activator significantly weakened the effects of si-CD147 on inhibiting the proliferation, migration, and invasion of A549 cells, while RAP1 inhibitor showed the opposite results (P < 0.001, Fig. 8A-C). Compared to the si-CD147 group, Rap1 activator significantly decreased apoptosis of A549 cells, while apoptosis of A549 cells was promoted in Rap1 inhibitor treated group (Fig. 8D). Similarly, Rap1 activator also significantly eliminated the role of si-CD147 on up-regulating FASN and ACOX1, and Rap1 inhibitor significantly reduced expression of FASN and ACOX1 compared with the si-CD147 group (P < 0.01, Fig. 8E).

## Discussion

Until now, molecular targeted therapy has achieved great advantages in the treatment of a myriad of cancer types [19]. Massive molecules have been proved to exhibit the potential in inhibiting the growth, progression, and metastasis of LUAD [20–23]. CD147 is a tumor-associated glycoprotein, which may also be a potential target for the treatment of LUAD. This research mainly focused on function and underlying action mechanisms of CD147 in LUAD. Our results showed an up-regulation of CD147 in LUAD cells. Silencing of CD147 inhibited the proliferation, migration, invasion, and lipid metabolism dysregulation of LUAD cells, as well as the growth of tumor xenografts in mice. In addition, the Rap1 signaling pathway was revealed as an action target of CD147 in LUAD.

CD147, also named as Bsg and EMMPRIN, is a transmembrane protein that widely expressed in a variety of cells, mainly including red cells, leucocytes, platelets, keratinocytes, and endothelial cells [24]. Via regulating T-cell activation, cell adhesion, cell metabolism, and extracellular matrix remodeling, etc., CD147 exerts multi-functions in various physiological and pathological processes, mainly including reproduction, neural function, inflammation, and cardiovascular diseases, etc. [4, 24, 25]. Notably, CD147 is closely involved in tumorigenesis and progression, and its overexpression has been shown to be a prognostic biomarker for more than 14 types of cancers in different organs, including the LUAD [5, 26]. Consistently, an up-regulation of CD147 in LUAD cells was revealed in this study. In addition, accumulating evidence has demonstrated that CD147 can enhance the proliferation, metastasis, glycolysis, and multi-drug resistance of cancer cells [27–29]. Herein, silencing of CD147 suppressed the viability, migration, invasion and promoted apoptosis of two LUAD cell lines, while overexpression of CD147 showed the opposite results. Our results were consistent with previous literatures, and indicated that CD147 was an oncogene in LUAD. Moreover, in vivo experiments showed that silencing of CD147 also inhibited the growth of tumor xenografts, which further illustrated the therapeutic potential of CD147 silencing against LUAD.

In addition to the conventional malignant characteristics, silencing of CD147 also decreased the expression of FASN and ACOX1 both in vitro and in vivo. Since FASN and ACOX1 are two key genes involved in lipid metabolism, our result indicated an inhibiting role of CD147 silencing in lipid metabolism in LUAD. In tumor microenvironment, lipid metabolism is usually enhanced to support the aggravated malignant features of cancer cells [15]. Targeting lipid metabolism has become an attractive choice for cancer treatment [30]. Emerging evidence has determined that many molecules exhibit therapeutic potential against LUAD through inhibiting lipid metabolism, such as Anlotinib (a vascular endothelial growth factor receptor inhibitor) [31] and endothelial lipase gene [32]. To combine with our findings, we speculated that the inhibition of lipid metabolism may contribute to the role of CD147 silencing in inhibiting LUAD.

In this study, the downstream molecular mechanisms of CD147 in LUAD were further explored. Until now, a variety of signaling pathways has been revealed as the downstream targets of CD147 in diverse cancers, such as the Akt/mTOR/SREBP1c pathway in hepatocellular carcinoma [17], β-TrCP/Nrf2 pathway in glioma [33], MEK pathway in hypopharyngeal squamous cell carcinoma [34], JAK/STAT pathway in breast cancer [35], and NF-kB pathway in head and neck squamous cell carcinoma [36]. In this research, total 1436 DEGs were screened by transcriptome sequencing, which were functionally enriched in diverse signaling pathways. The Rap1 signaling pathway closely associated with tumorigenesis was then selected as a research target. As a key regulator in cell adhesion and integrin function, Rap1 can initiate and sustain the ERK signaling to promote cancer progression [37]. Previous researches have confirmed the Rap1 signaling is activated in various malignancies, including prostate cancer [38], breast cancer [39], ovarian cancer [40], colon cancer [41], NSCLC [42], etc. In this study, silencing of CD147 led to the blocking of the Rap1 signaling pathway in LUAD cells, evidenced by the down-regulation of Rap1 and p-p38 MAPK/p38 MAPK. Jin et al. have shown that RBM10 suppresses the proliferation of LUAD cells through inhibiting the RAP1/AKT/CREB signaling pathway [43]. Similarly, GREM1 enhances the migration, invasion, and epithelial-mesenchymal transition of NSCLC cells through activating the Rap1 pathway [14]. Therefore, the blocking of the Rap1 pathway may make a great contribution to the anti-tumor outcomes. Herein, a Rap1 activator was further applied to confirm the regulatory relationship between CD147 and the Rap1 signaling pathway. As expected, Rap1 activator reversed the role of CD147 silencing on inhibiting the proliferation, migration, invasion, lipid metabolism and promoting apoptosis of A549 cells, and RAP1 inhibitor showed the opposite results. These phenomena further illustrated that silencing of CD147 may suppress the progression of LUAD via blocking the Rap1 signaling pathway.

In fact, this study also revealed many other signaling pathways that may also participated in the action mechanisms of CD147 in LUAD. Many hub genes discovered in the PPI network may also be the potential downstream targets of CD147. Further researches on more detailed mechanisms are still required. However, the findings of this study have some limitations. In this study, the investigation of CD147/Rap1 on lipid metabolism dysregulation is limited. Furthermore, the exact mechanism of Rap1 in LUAD needs to be further studied. In the future, the research on the effect of CD147/Rap1 on lipid metabolism in LUAD can be deeply excavated.

## Conclusions

In conclusion, there was an up-regulation of CD147 in LUAD cells. Silencing of CD147 suppressed the proliferation, migration, invasion, and lipid metabolism dysregulation of LUAD cells, as well as the tumor xenograft growth in vivo. The Rap1 signaling pathway was a downstream target of CD147, and its blocking contributed to the anti-tumor outcomes in LUAD.

### Electronic supplementary material

Below is the link to the electronic supplementary material.

Supplementary Material 1

## Data Availability

All data in the manuscript is available through the responsible corresponding author.

## References

[1] Relli V, Trerotola M, Guerra E, Alberti S (2019). Abandoning the notion of Non-Small Cell Lung Cancer. Trends Mol Med.

[2] Gridelli C, Rossi A, Carbone DP, Guarize J, Karachaliou N, Mok T, Petrella F, Spaggiari L, Rosell R (2015). Non-small-cell lung cancer. Nat Rev Dis Primers.

[3] Network TCGAR (2014). Comprehensive molecular profiling of lung adenocarcinoma. Nature.

[4] Guindolet D, Gabison EE (2020). Role of CD147 (EMMPRIN/Basigin) in tissue remodeling. Anat Rec (Hoboken).

[5] Landras A, Reger de Moura C, Jouenne F, Lebbe C, Menashi S, Mourah S. CD147 is a Promising Target of Tumor Progression and a prognostic biomarker. Cancers (Basel) 2019, 11. 10.3390/cancers11111803 PMC6896083 31744072

[6] Wang H, Zhuo Y, Hu X, Shen W, Zhang Y, Chu T (2015). CD147 deficiency blocks IL-8 secretion and inhibits lung cancer-induced osteoclastogenesis. Biochem Biophys Res Commun.

[7] Zhang X, Tian T, Liu C, Fang X (2017). Elevated CD147 expression is associated with shorter overall survival in non-small cell lung cancer. Oncotarget.

[8] Zeng HZ, Qu YQ, Liang AB, Deng AM, Zhang WJ, Xiu B, Wang H (2011). Expression of CD147 in advanced non-small cell lung cancer correlated with cisplatin-based chemotherapy resistance. Neoplasma.

[9] Wang S, Li B, Wang S, Li Y, Li J (2011). Expression and clinical significance of CD147 and MMP-2 in squamous cell carcinoma and adenocarcinoma of the lungs. Zhongguo Fei Ai Za Zhi.

[10] Zhang Y, Liu J, Sun Y, Yu X, Wang J, Dai D, Zhu Y, Song X, Zhu L, Li X, Xu W (2020). Enhanced glucose metabolism mediated by CD147 is associated with (18) F-FDG PET/CT imaging in lung adenocarcinoma. Thorac Cancer.

[11] Chrzanowska-Wodnicka M (2017). Rap1 in endothelial biology. Curr Opin Hematol.

[12] Deregowska A, Wnuk M. RAP1/TERF2IP-A multifunctional player in Cancer Development. Cancers (Basel) 2021, 13. 10.3390/cancers13235970 PMC8657031 34885080

[13] Li H, Liang J, Wang J, Han J, Li S, Huang K, Liu C (2021). Mex3a promotes oncogenesis through the RAP1/MAPK signaling pathway in colorectal cancer and is inhibited by hsa-miR-6887-3p. Cancer Commun (Lond).

[14] Kan J, Fu B, Zhou R, Zhou D, Huang Y, Zhao H, Zhang Y, Rong Y, Dong J, Xia L (2022). He-Chan Pian inhibits the metastasis of non-small cell lung cancer via the mir-205-5p-mediated regulation of the GREM1/Rap1 signaling pathway. Phytomedicine.

[15] Bian X, Liu R, Meng Y, Xing D, Xu D, Lu Z. Lipid metabolism and cancer. J Exp Med 2021, 218. 10.1084/jem.20201606 PMC7754673 33601415

[16] Cheng C, Geng F, Cheng X, Guo D (2018). Lipid metabolism reprogramming and its potential targets in cancer. Cancer Commun (Lond).

[17] Li J, Huang Q, Long X, Zhang J, Huang X, Aa J, Yang H, Chen Z, Xing J (2015). CD147 reprograms fatty acid metabolism in hepatocellular carcinoma cells through Akt/mTOR/SREBP1c and P38/PPARalpha pathways. J Hepatol.

[18] Sun C, Gao W, Liu J, Cheng H, Hao J (2020). FGL1 regulates acquired resistance to Gefitinib by inhibiting apoptosis in non-small cell lung cancer. Respir Res.

[19] Lee YT, Tan YJ, Oon CE (2018). Molecular targeted therapy: treating cancer with specificity. Eur J Pharmacol.

[20] Xie X, Cai X, Zhou F, Li Y, Liu Q, Cai L, Zhu W, Wei J, Jin C, Liu Z (2022). GPR37 promotes cancer growth by binding to CDK6 and represents a new theranostic target in lung adenocarcinoma. Pharmacol Res.

[21] He M, Han Y, Cai C, Liu P, Chen Y, Shen H, Xu X, Zeng S (2021). CLEC10A is a prognostic biomarker and correlated with clinical pathologic features and immune infiltrates in lung adenocarcinoma. J Cell Mol Med.

[22] Song H, Liu D, Wang L, Liu K, Chen C, Wang L, Ren Y, Ju B, Zhong F, Jiang X (2022). Methyltransferase like 7B is a potential therapeutic target for reversing EGFR-TKIs resistance in lung adenocarcinoma. Mol Cancer.

[23] Wang J, Liu J, Hou Q, Xu M (2022). LINC02126 is a potential diagnostic, prognostic and immunotherapeutic target for lung adenocarcinoma. BMC Pulm Med.

[24] Pennings GJ, Kritharides L (2014). CD147 in cardiovascular disease and thrombosis. Semin Thromb Hemost.

[25] Muramatsu T, Miyauchi T (2003). Basigin (CD147): a multifunctional transmembrane protein involved in reproduction, neural function, inflammation and tumor invasion. Histol Histopathol.

[26] Zhong X, Li M, Nie B, Wu F, Zhang L, Wang E, Han Y (2013). Overexpressions of RACK1 and CD147 associated with poor prognosis in stage T1 pulmonary adenocarcinoma. Ann Surg Oncol.

[27] Xiong L, Edwards CK 3rd, Zhou L. The biological function and clinical utilization of CD147 in human diseases: a review of the current scientific literature. Int J Mol Sci. 2014;15:17411–41. 10.3390/ijms151017411 PMC4227170 25268615

[28] Kanekura T, Chen X (2010). CD147/basigin promotes progression of malignant melanoma and other cancers. J Dermatol Sci.

[29] Lian C, Guo Y, Zhang J, Chen X, Peng C (2017). Targeting CD147 is a Novel Strategy for Antitumor Therapy. Curr Pharm Des.

[30] Broadfield LA, Pane AA, Talebi A, Swinnen JV, Fendt SM (2021). Lipid metabolism in cancer: new perspectives and emerging mechanisms. Dev Cell.

[31] Shen J, Huang J, Huang Y, Chen Y, Li J, Luo P, Zhang Q, Qiu Y, Wang L, Jiang H (2022). Anlotinib suppresses lung adenocarcinoma growth via inhibiting FASN-mediated lipid metabolism. Ann Transl Med.

[32] Wang S, Chen Z, Lv H, Wang C, Wei H, Yu J (2023). LIPG is a novel prognostic biomarker and correlated with immune infiltrates in lung adenocarcinoma. J Clin Lab Anal.

[33] Bu X, Qu X, Guo K, Meng X, Yang X, Huang Q, Dou W, Feng L, Wei X, Gao J (2021). CD147 confers temozolomide resistance of glioma cells via the regulation of beta-TrCP/Nrf2 pathway. Int J Biol Sci.

[34] Suzuki S, Toyoma S, Kawasaki Y, Nanjo H, Yamada T (2021). CD147 promotes invasion and MMP-9 expression through MEK signaling and predicts poor prognosis in hypopharyngeal squamous cell carcinoma. Adv Clin Exp Med.

[35] Knutti N, Huber O, Friedrich K (2019). CD147 (EMMPRIN) controls malignant properties of breast cancer cells by interdependent signaling of wnt and JAK/STAT pathways. Mol Cell Biochem.

[36] Yu B, Zhang Y, Wu K, Wang L, Jiang Y, Chen W, Yan M (2019). CD147 promotes progression of head and neck squamous cell carcinoma via NF-kappa B signaling. J Cell Mol Med.

[37] Shah S, Brock EJ, Ji K, Mattingly RR (2019). Ras and Rap1: a tale of two GTPases. Semin Cancer Biol.

[38] Bailey CL, Kelly P, Casey PJ (2009). Activation of Rap1 promotes prostate cancer metastasis. Cancer Res.

[39] McSherry EA, Brennan K, Hudson L, Hill AD, Hopkins AM (2011). Breast cancer cell migration is regulated through junctional adhesion molecule-A-mediated activation of Rap1 GTPase. Breast Cancer Res.

[40] Che YL, Luo SJ, Li G, Cheng M, Gao YM, Li XM, Dai JM, He H, Wang J, Peng HJ (2015). The C3G/Rap1 pathway promotes secretion of MMP-2 and MMP-9 and is involved in serous ovarian cancer metastasis. Cancer Lett.

[41] Ramsey A, Akana L, Miyajima E, Douglas S, Gray J, Rowland A, Sharma KD, Xu J, Xie JY, Zhou GL (2023). CAP1 (cyclase-associated protein 1) mediates the cyclic AMP signals that activate Rap1 in stimulating matrix adhesion of colon cancer cells. Cell Signal.

[42] Tan HY, Ho VW, Chan YT, Zhang C, Wang N, Xia W, Feng Y (2020). Combination of Gentiana rhodantha and Gerbera anandria in the BL02 formula as therapeutics to non-small cell lung carcinoma acting via Rap1/cdc42 signaling: a transcriptomics/ bio-informatics biological validation approach. Pharmacol Res.

[43] Jin X, Di X, Wang R, Ma H, Tian C, Zhao M, Cong S, Liu J, Li R, Wang K (2019). RBM10 inhibits cell proliferation of lung adenocarcinoma via RAP1/AKT/CREB signalling pathway. J Cell Mol Med.

