# Correction: Silencing of CD147 inhibits cell proliferation, migration, invasion, lipid metabolism dysregulation and promotes apoptosis in lung adenocarcinoma via blocking the Rap1 signaling pathway

**DOI:** 10.1186/s12931-024-02711-7

**Published:** 2024-03-21

**Authors:** Ning Zhang, Zhouzhong Liu, Xuwang Lai, Shubin Liu, Yuli Wang

**Affiliations:** 1 Department of Gastroenterology, Ganzhou People’s Hospital, The Affiliated Ganzhou Hospital of Nanchang University, Ganzhou, 341000 Jiangxi China; 2 Department of Oncology, Ganzhou People’s Hospital, The Affiliated Ganzhou Hospital of Nanchang University, Ganzhou, 341000 Jiangxi China

**Correction** **:** **Respiratory Research (2023) 24:253** 10.1186/s12931-023-02532-0

Following publication of the original article [1], the authors identified that Figs. 1 and 2 were inadvertently swapped.

**Fig. 1:**
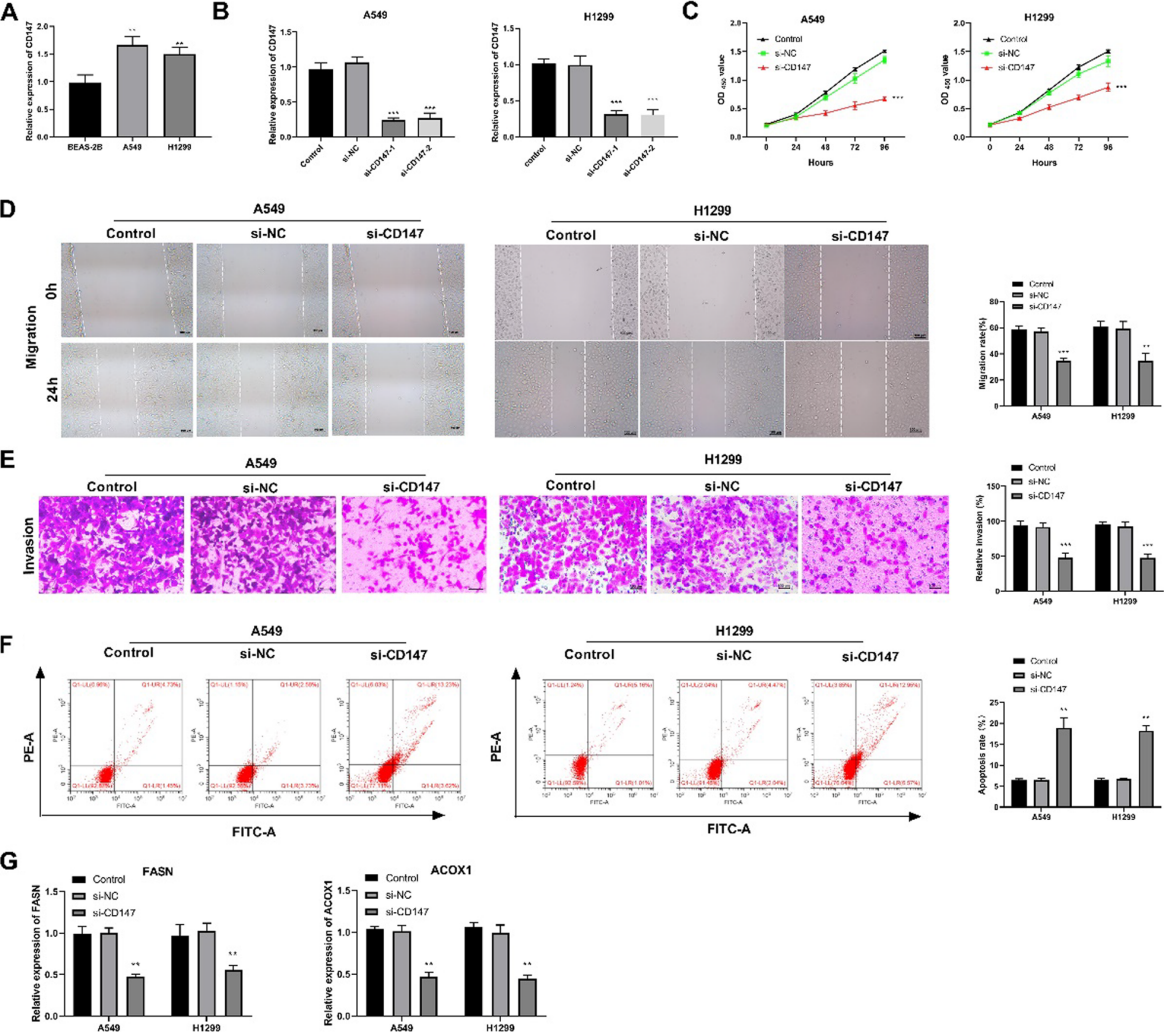
Silencing of CD147 inhibited the proliferation, migration, invasion, and lipid metabolism of LUAD cells. **A** The mRNA expression of CD147 in two LUAD cell lines (A549 and H1299 cells) and a normal human lung epithelial cell line (BEAS-2B cells) was detected by qRT-PCR; **p < 0.01 vs. BEAS-B cells. **B** The silencing efficiency of si-CD147-1/2 in LUAD cells was detected by qRT-PCR. **C**–**F** The viability, migration, invasion, and apoptosis of transfected LUAD cells was detected by CCK8 assay, wound healing assay, transwell assay, and flow cytometry, respectively. **G** The protein expression of FASN and ACOX1, two key genes involved in lipid metabolism was detected by western blot. *p < 0.05, **p < 0.01, ***p < 0.001 vs. Control

**Fig. 2:**
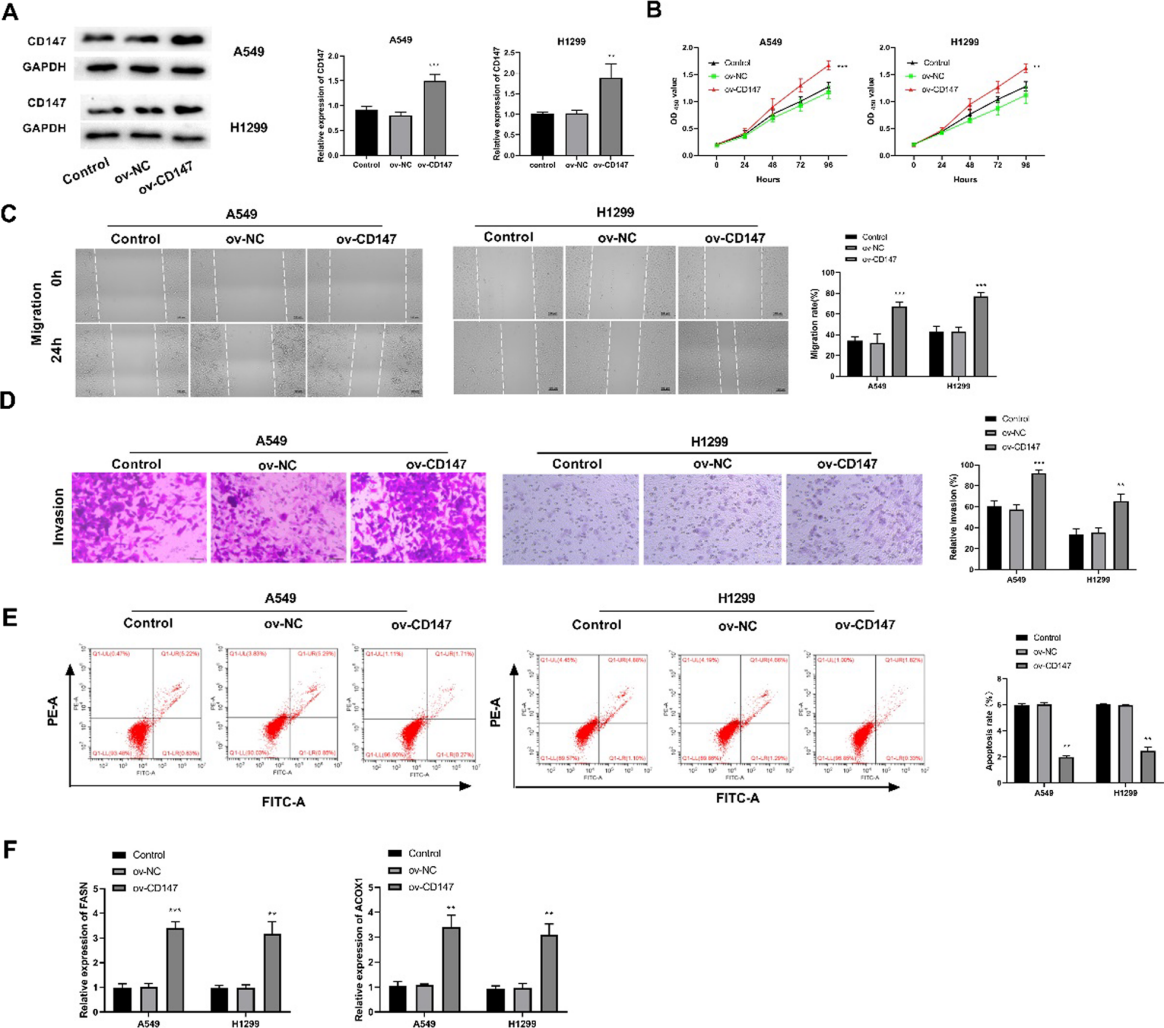
Overexpression of CD147 promoted the proliferation, migration, invasion, lipid metabolism of LUAD cells. **A** The protein and mRNA expression of CD147 in A549 and H1299 cells was detected by western blot and qRT-PCR; **p < 0.01 vs. control. **B**–**E** The viability, migration, invasion, and apoptosis of transfected LUAD cells was detected by CCK8 assay, wound healing assay, transwell assay, and flow cytometry, respectively. **F** The protein expression of FASN and ACOX1, two key genes involved in lipid metabolism was detected by western blot. *p < 0.05, **p < 0.01, ***p < 0.001 vs. Control

That is, Fig. 1 in the article should be changed to Fig. 2, and Fig. 2 in the article should be changed into Fig. 1, with no change in the position of figure notes.

These corrections do not significantly impact the overall findings and conclusions of the paper. The authors apologies for this error and assure all readers that the corrections do not alter the interpretations or validity of the research.

The correct figures and captions have been included in this correction, and the original article has been corrected.
