# Supplementary material for: Silencing of CD147 inhibits cell proliferation, migration, invasion, lipid metabolism dysregulation and promotes apoptosis in lung adenocarcinoma via blocking the Rap1 signaling pathway
Source: Respir Res. 2023 Oct 25;24:253. doi: 10.1186/s12931-023-02532-0 (PMC10601207; doi:10.1186/s12931-023-02532-0)
Supplement: Supplementary file 1 — Supplementary Material 1 [file 12931_2023_2532_MOESM1_ESM.pdf]

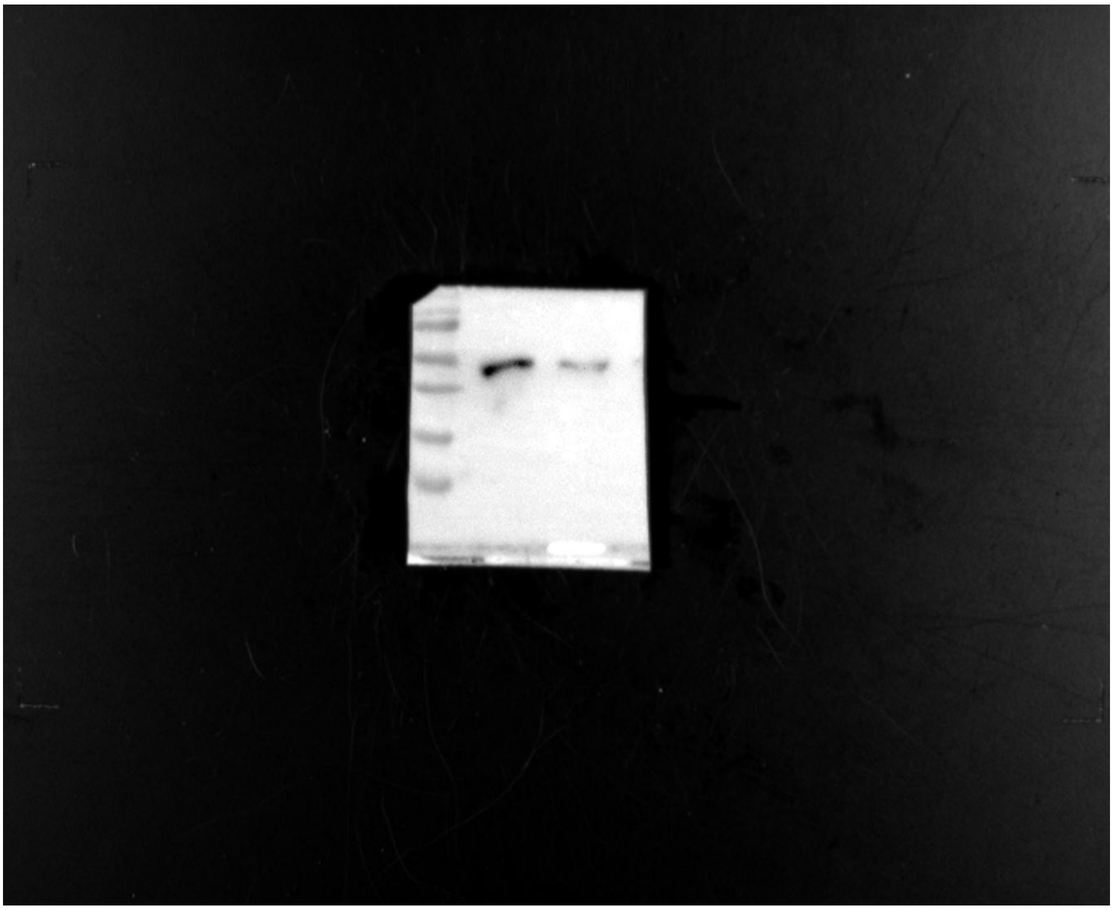

fig.2 ACOX1-1

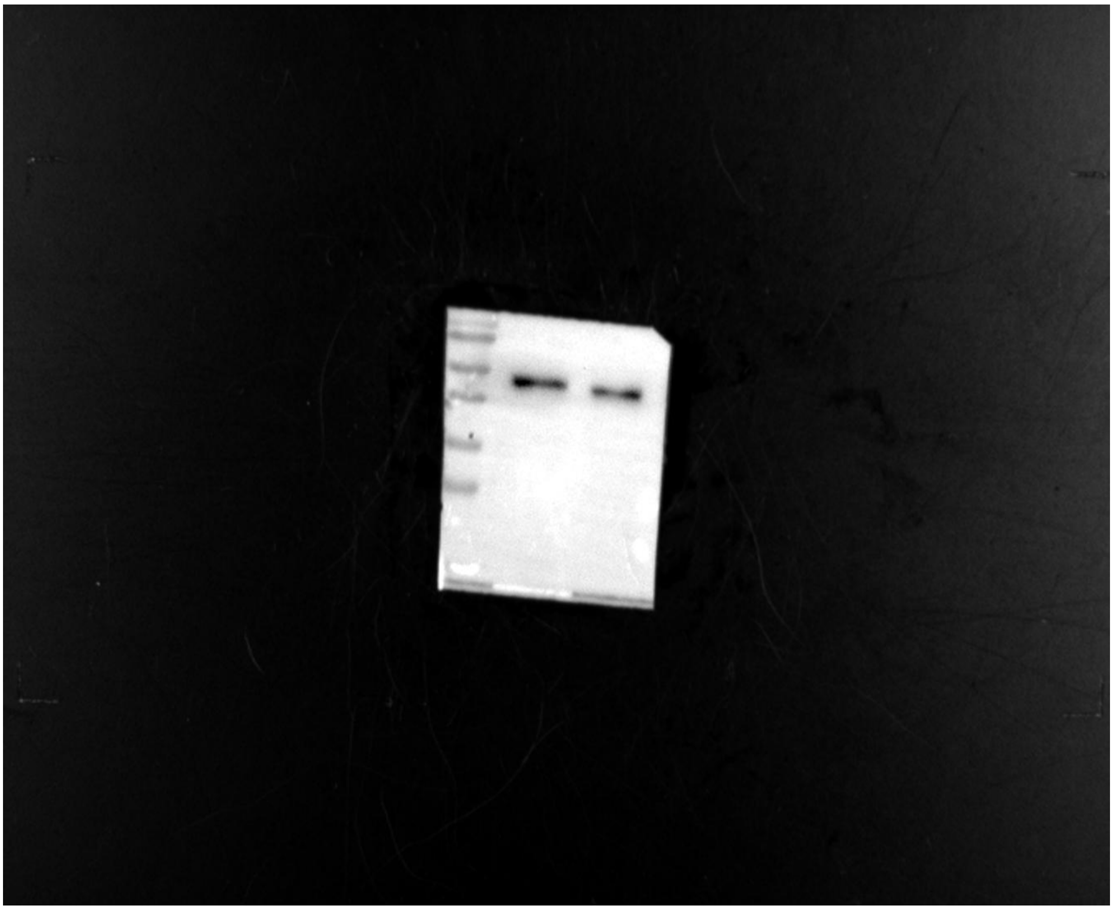

fig.2 ACOX1-1

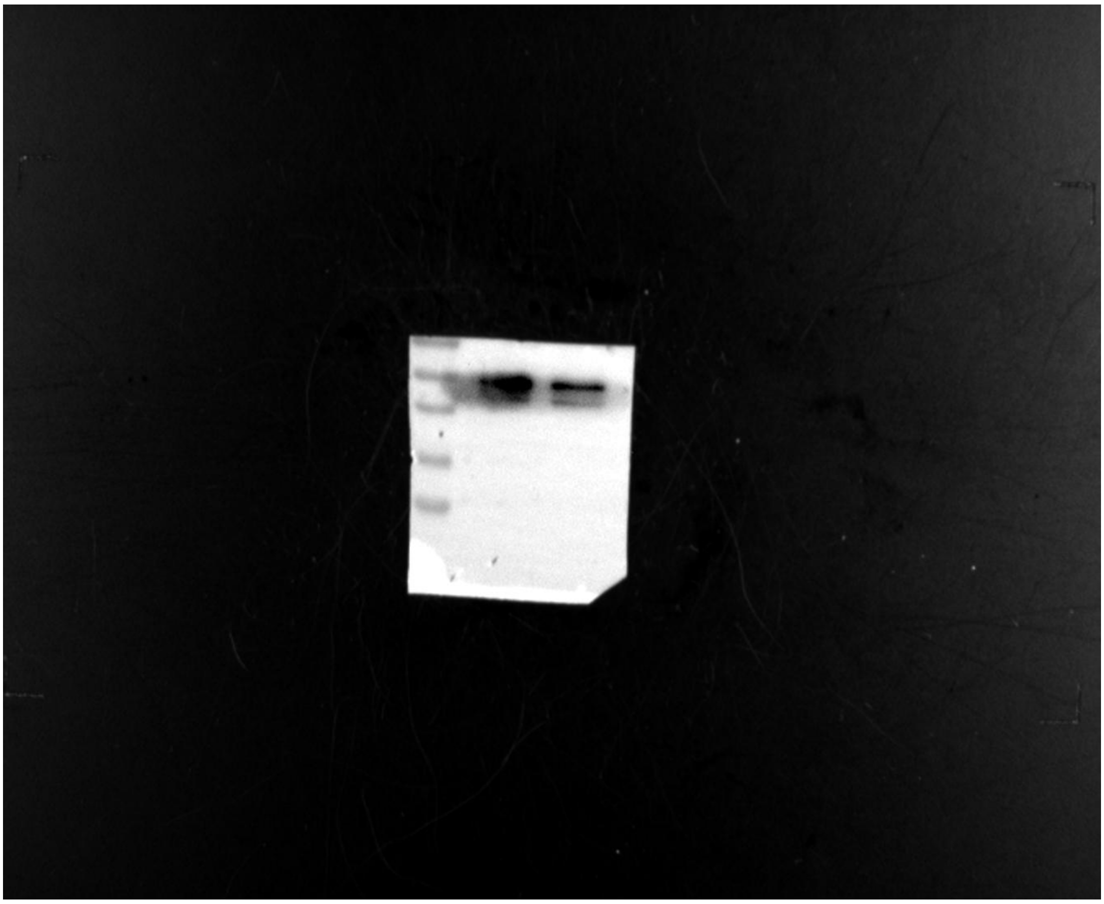

fig.2 ACOX1-3

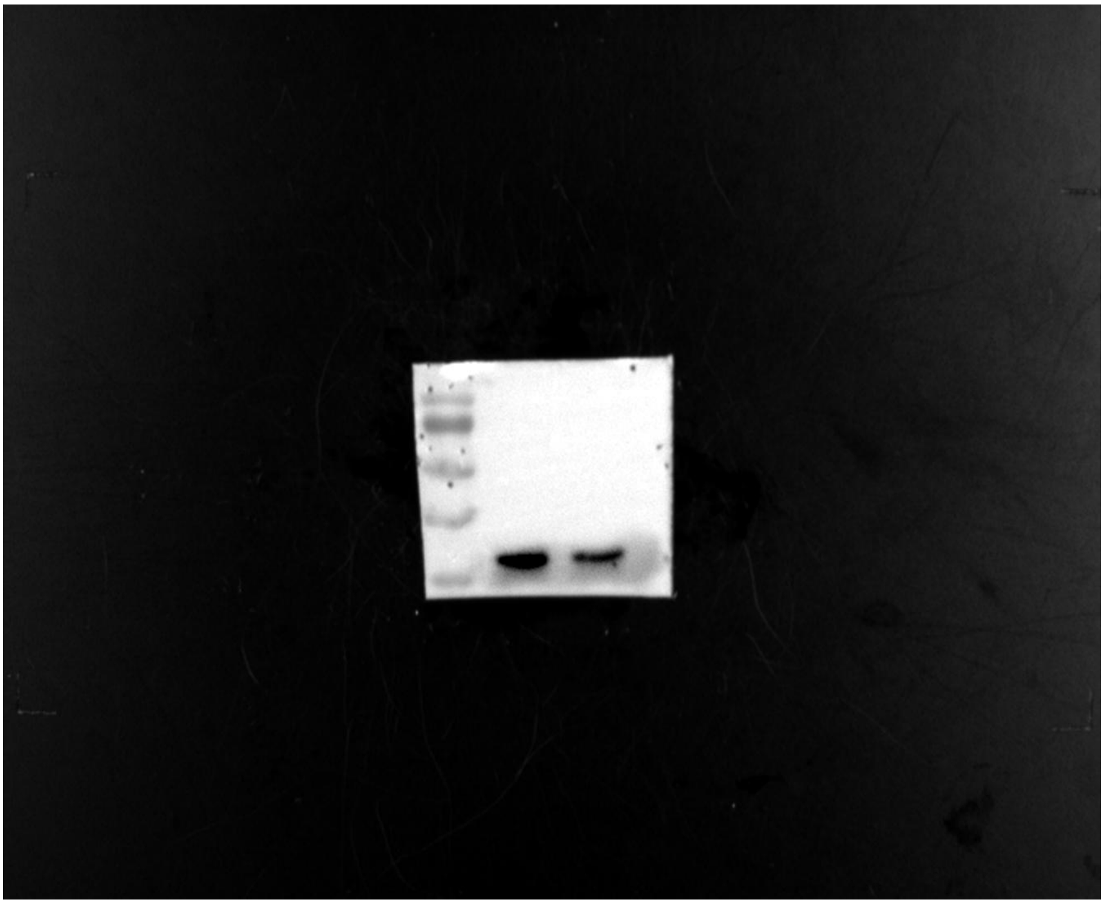

fig.2 CD147-1

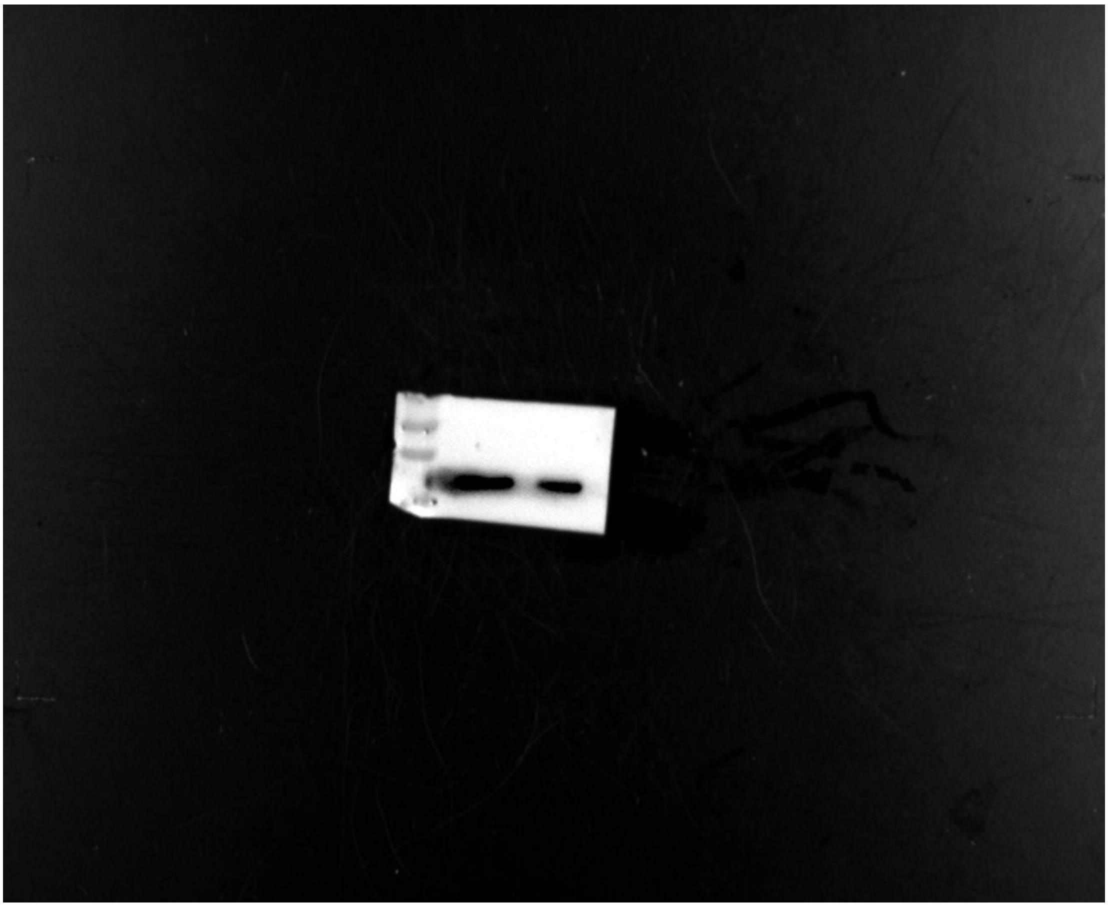

fig.2 CD147-2

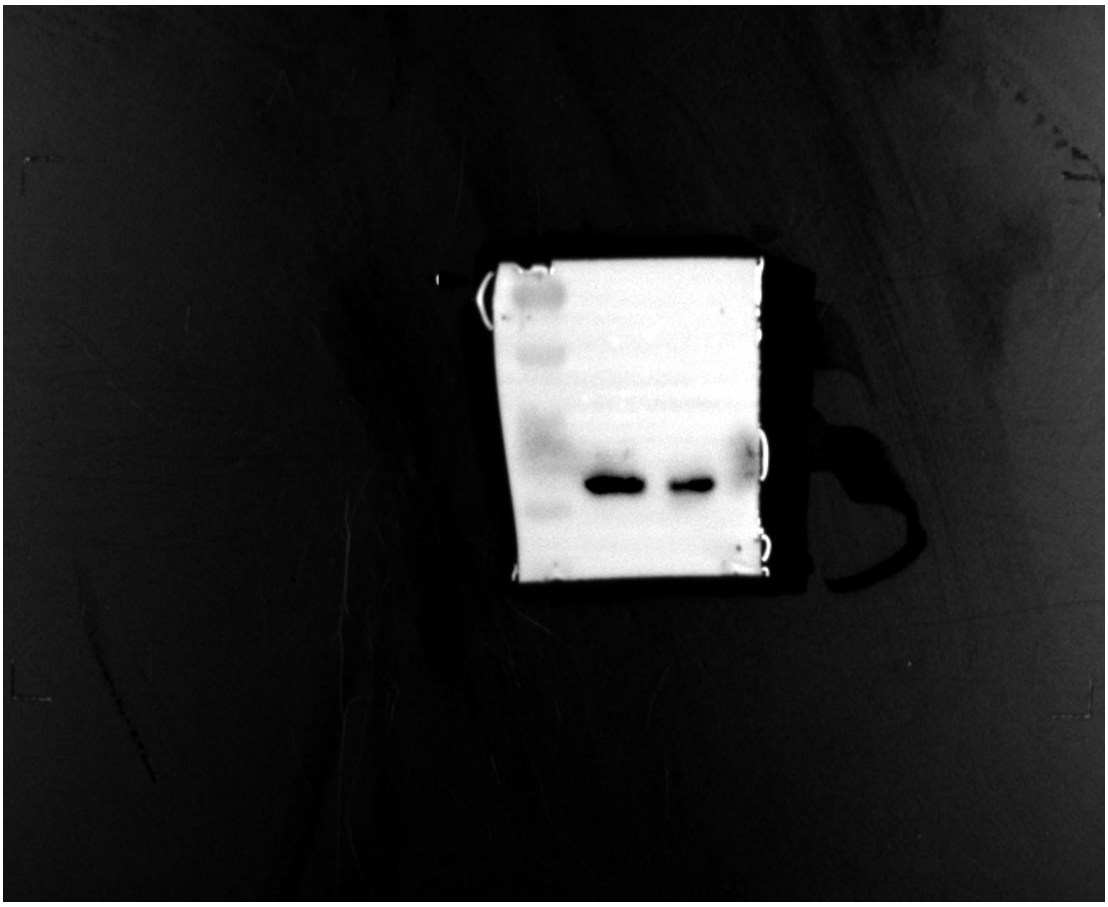

fig.2 CD147-3

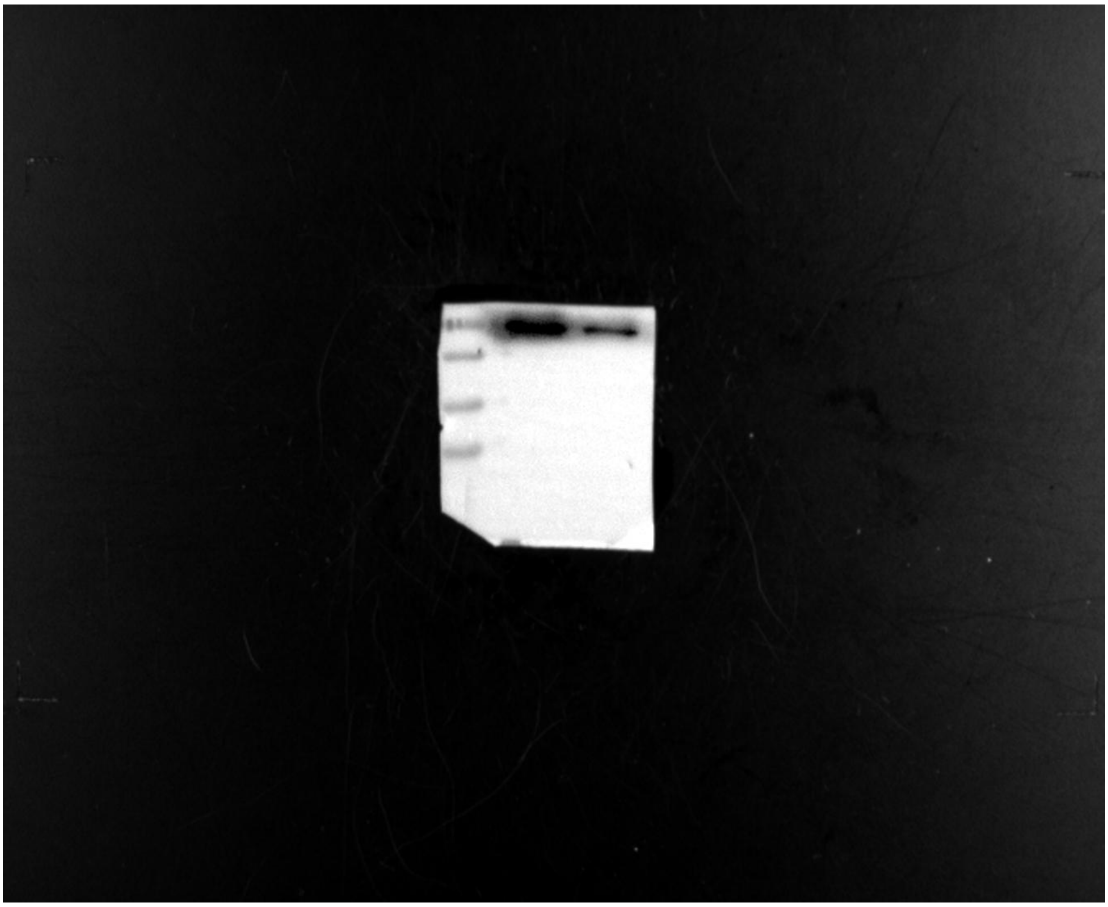

fig.2 FASN-1

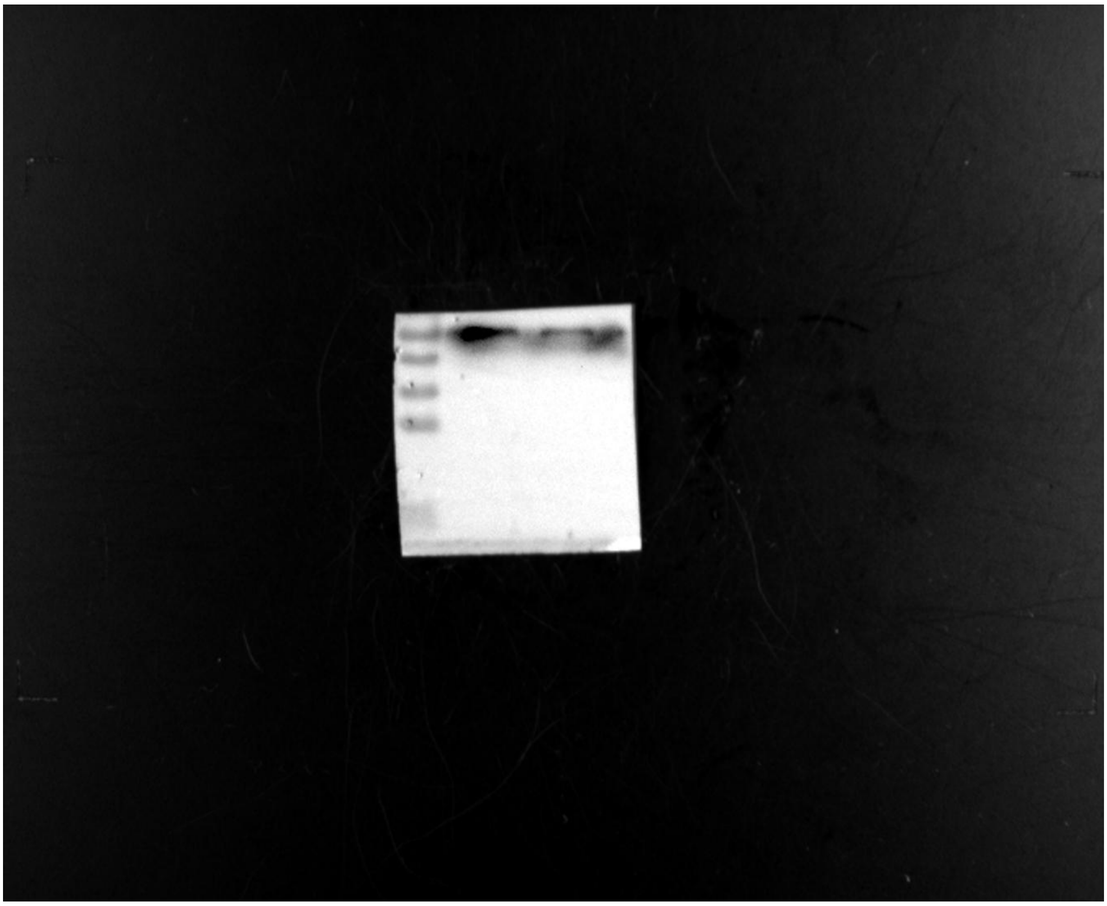

fig.2 FASN-2

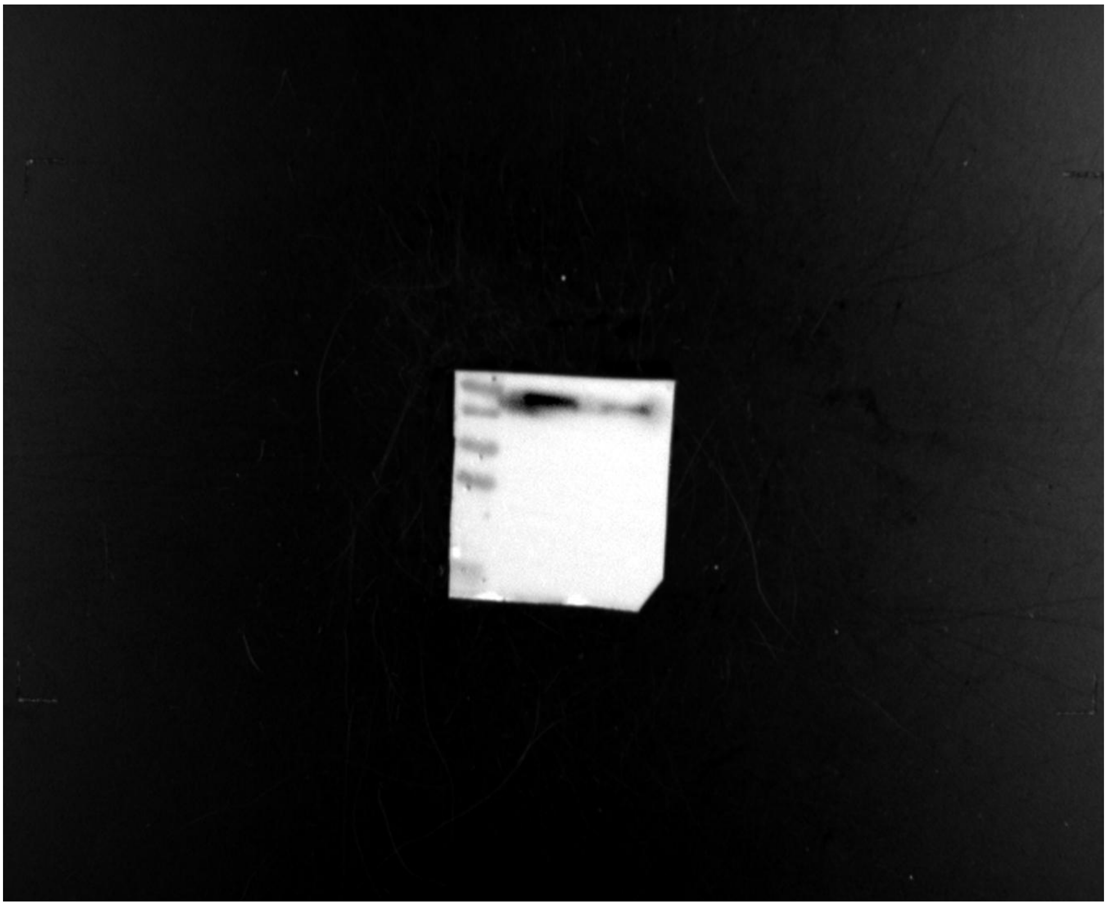

fig.2 FASN-3

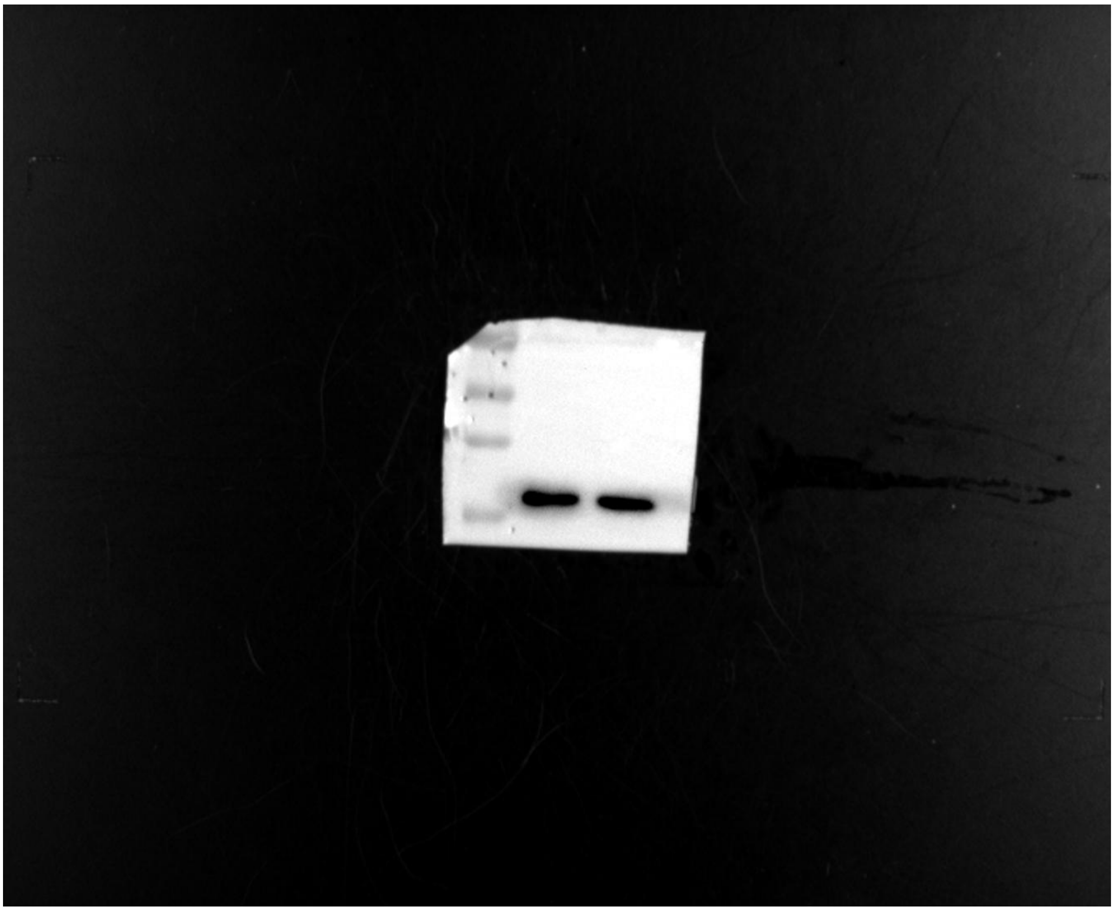

fig.2 GAPDH-1

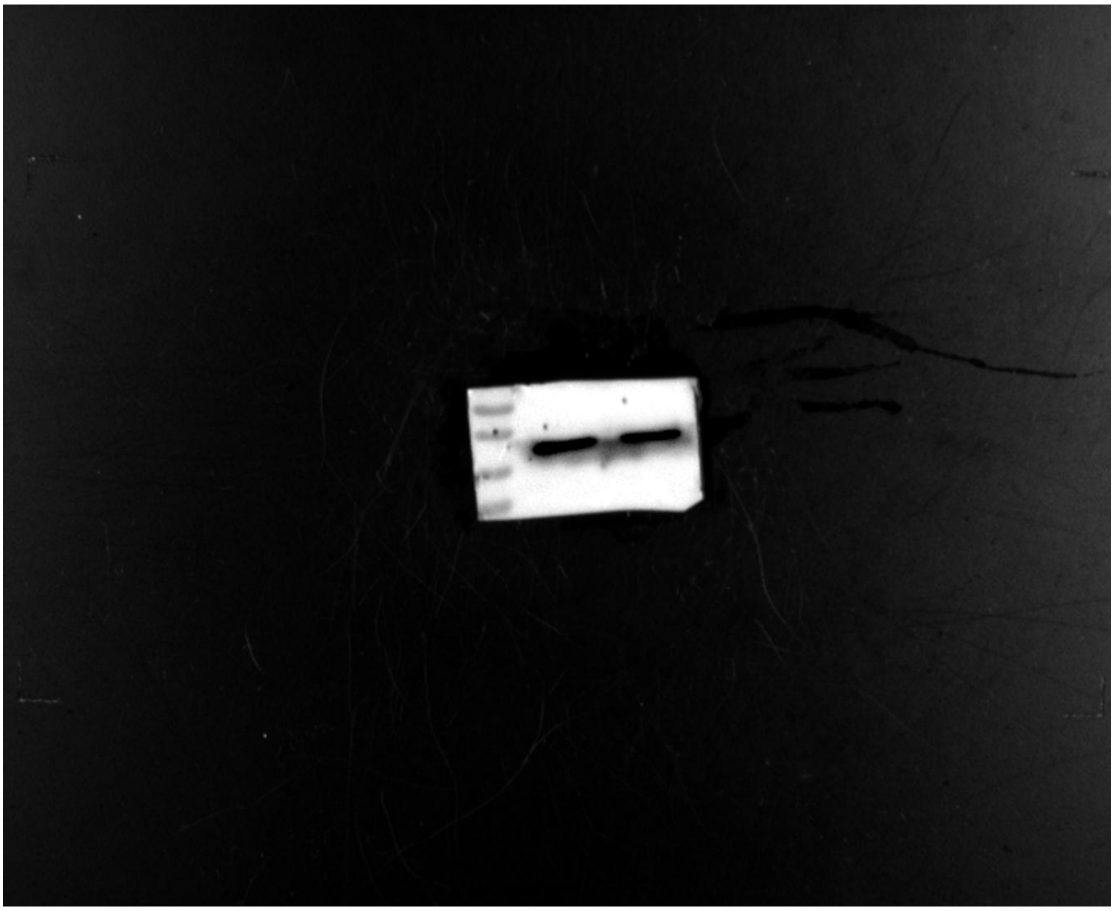

fig.2 GAPDH-2

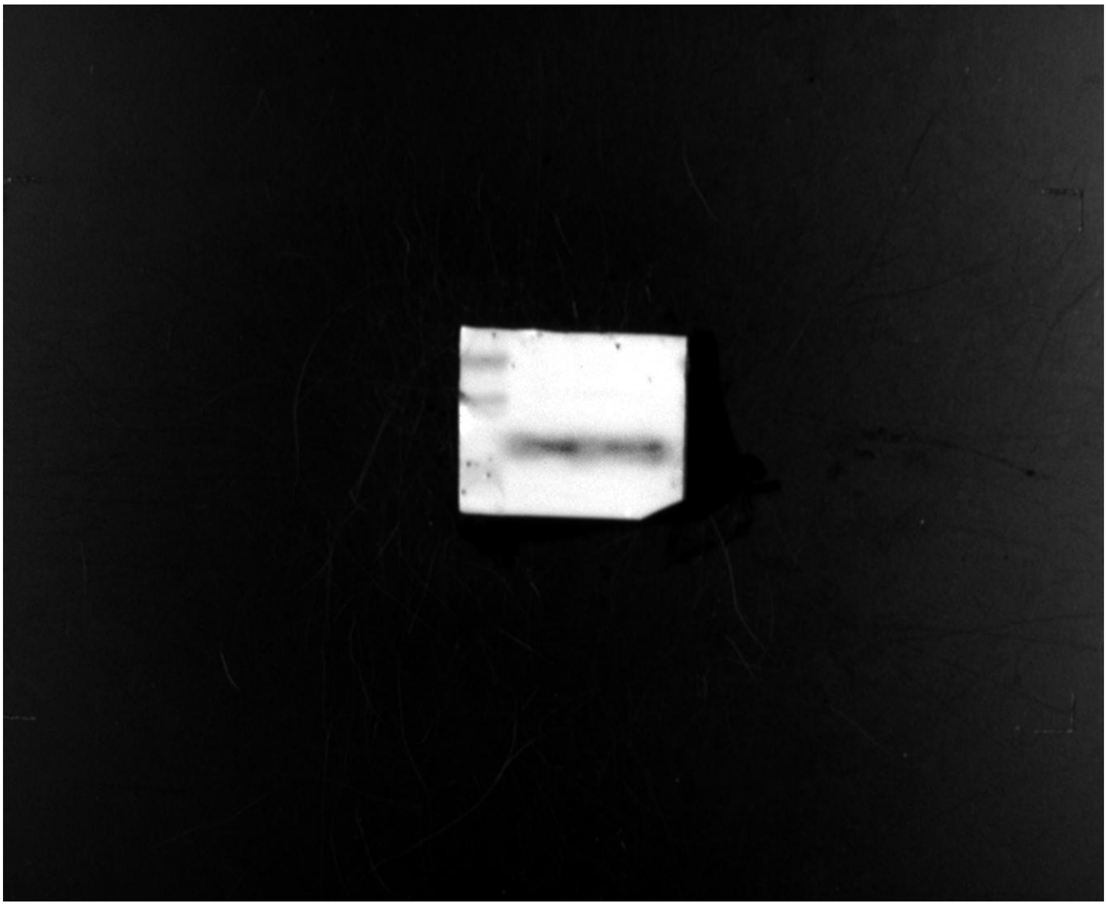

fig.2 GAPDH-3

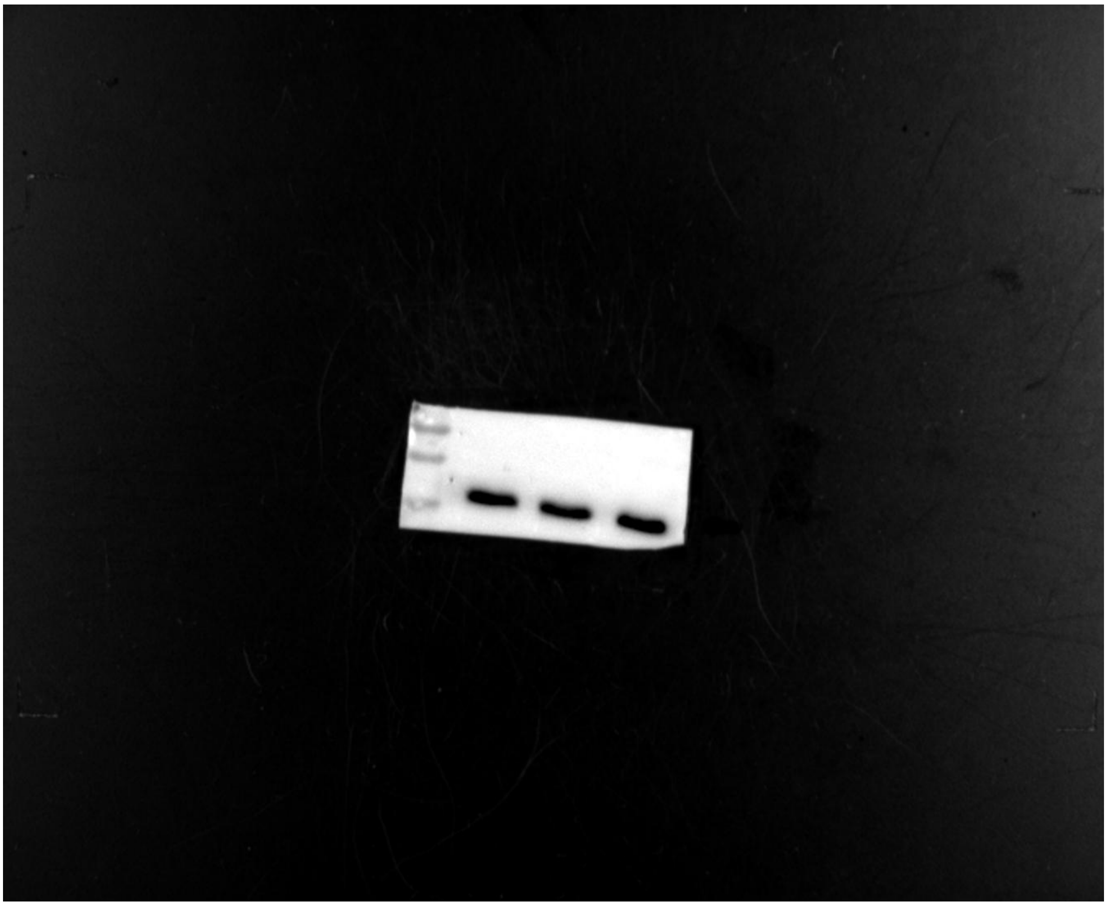

fig.6 GAPDH-1

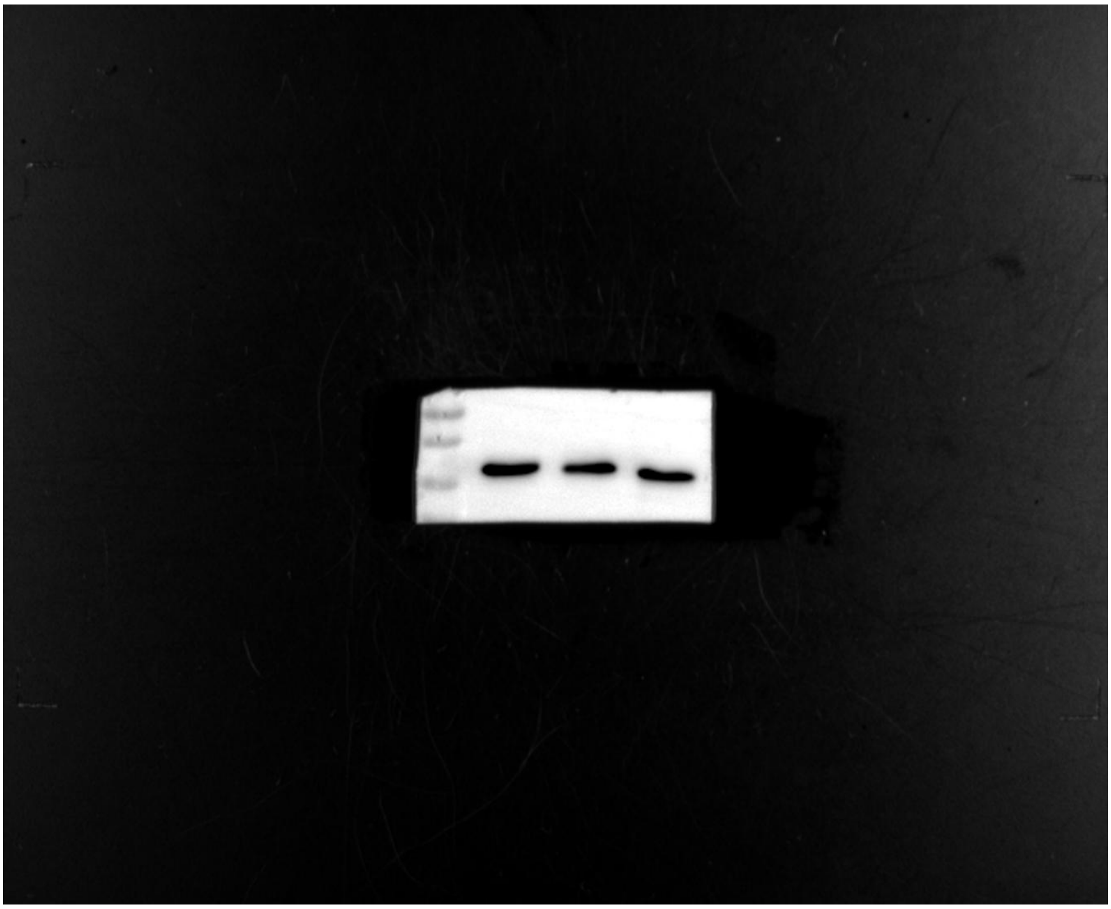

fig.6 GAPDH-2

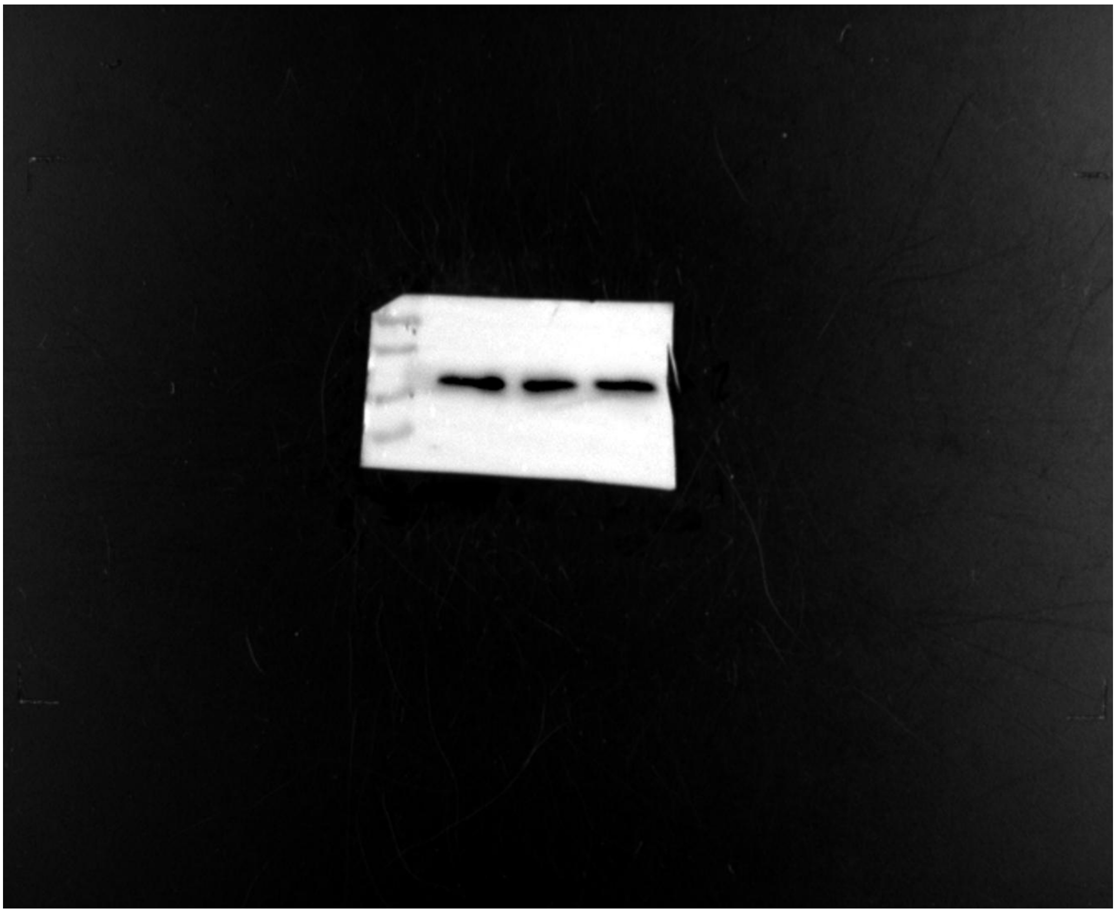

fig.6 GAPDH-3

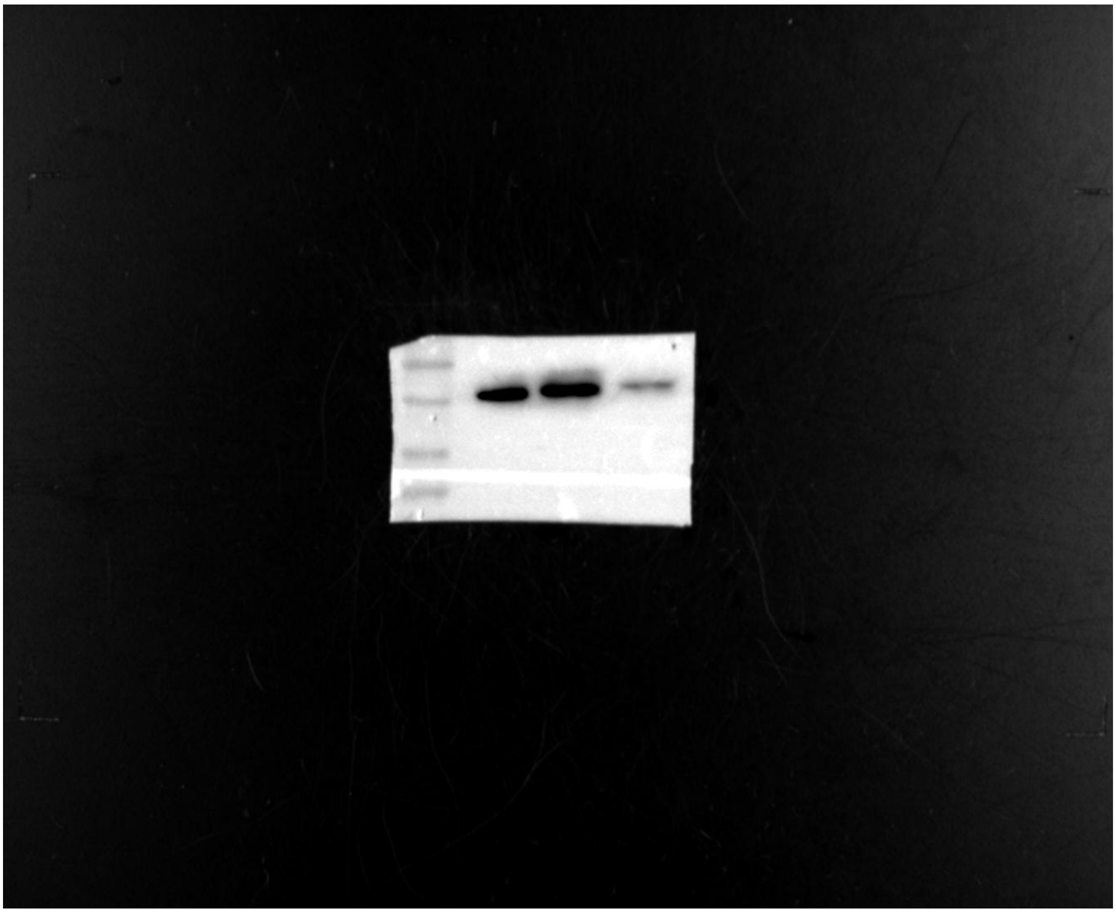

fig.6 RAP1-1

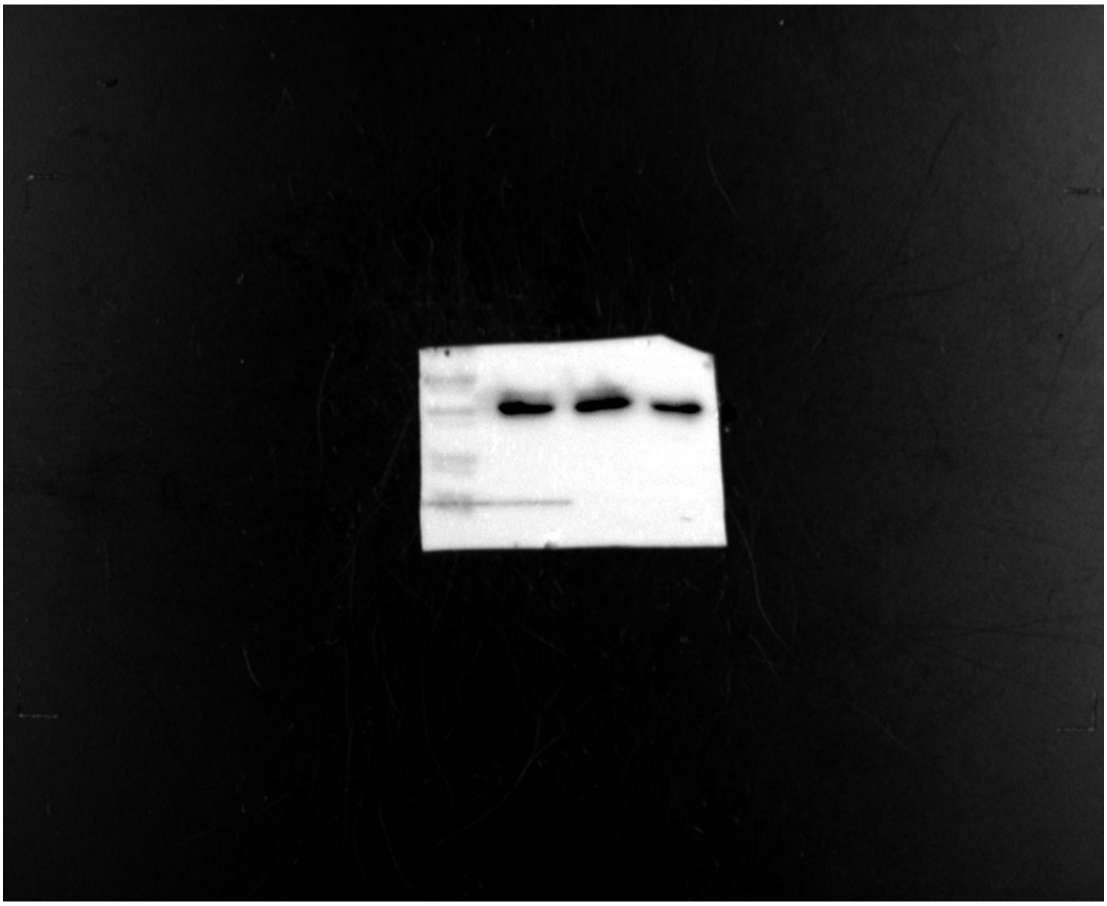

fig.6 RAP1-2

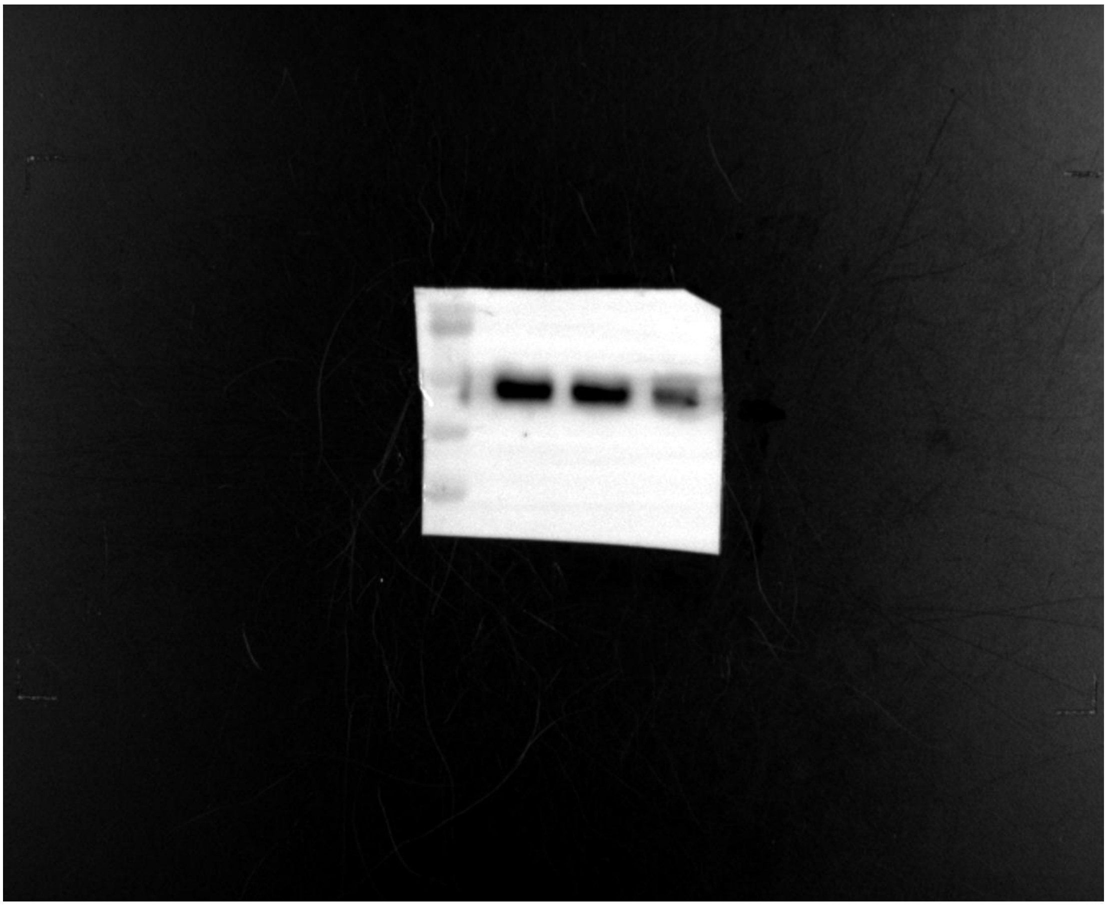

fig.6   RAP1-3

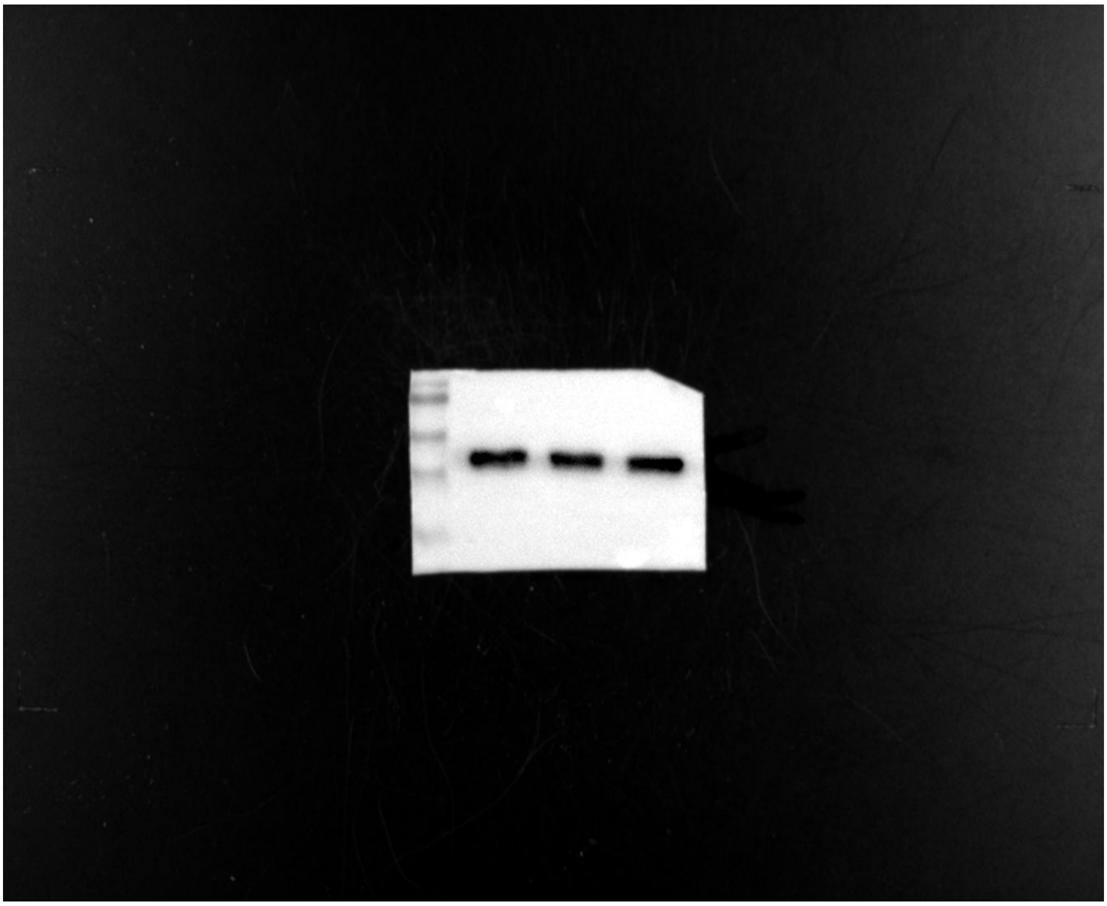

fig.6 P38 MAPK-1

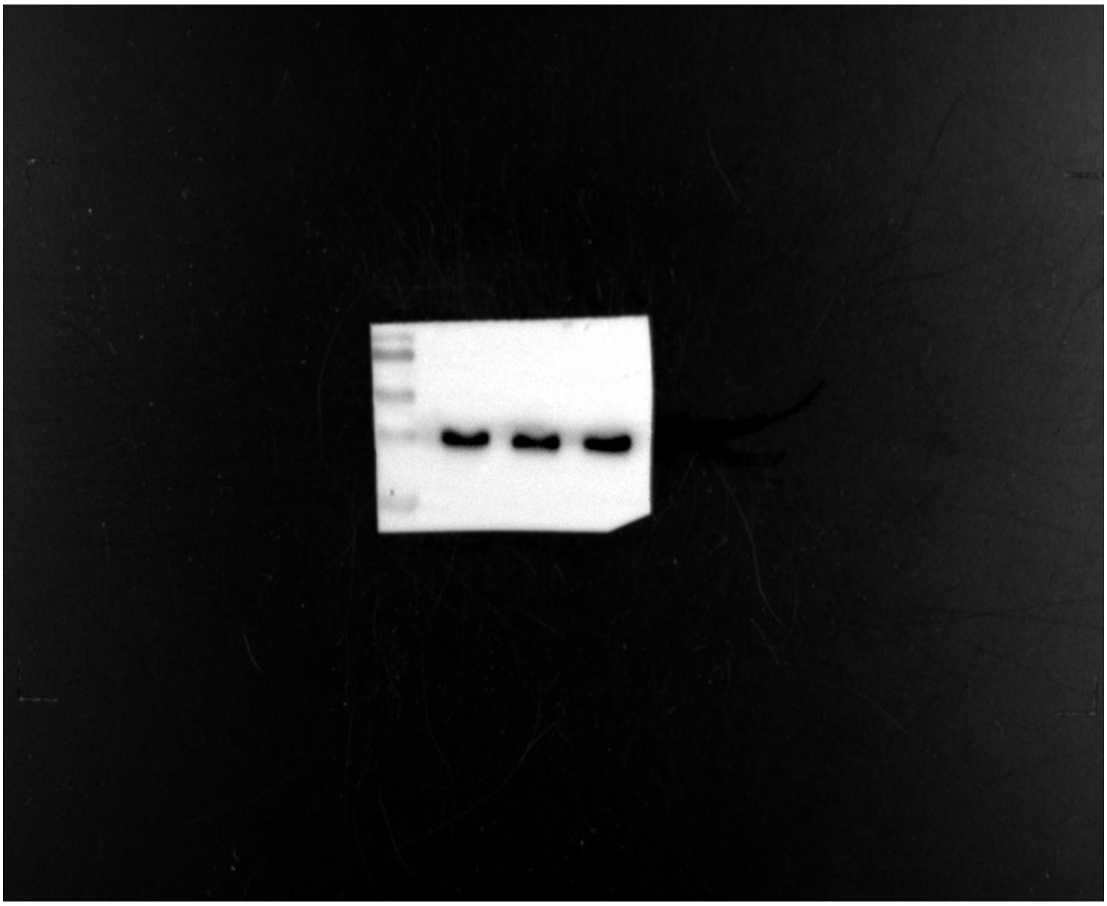

fig.6 P38 MAPK-2

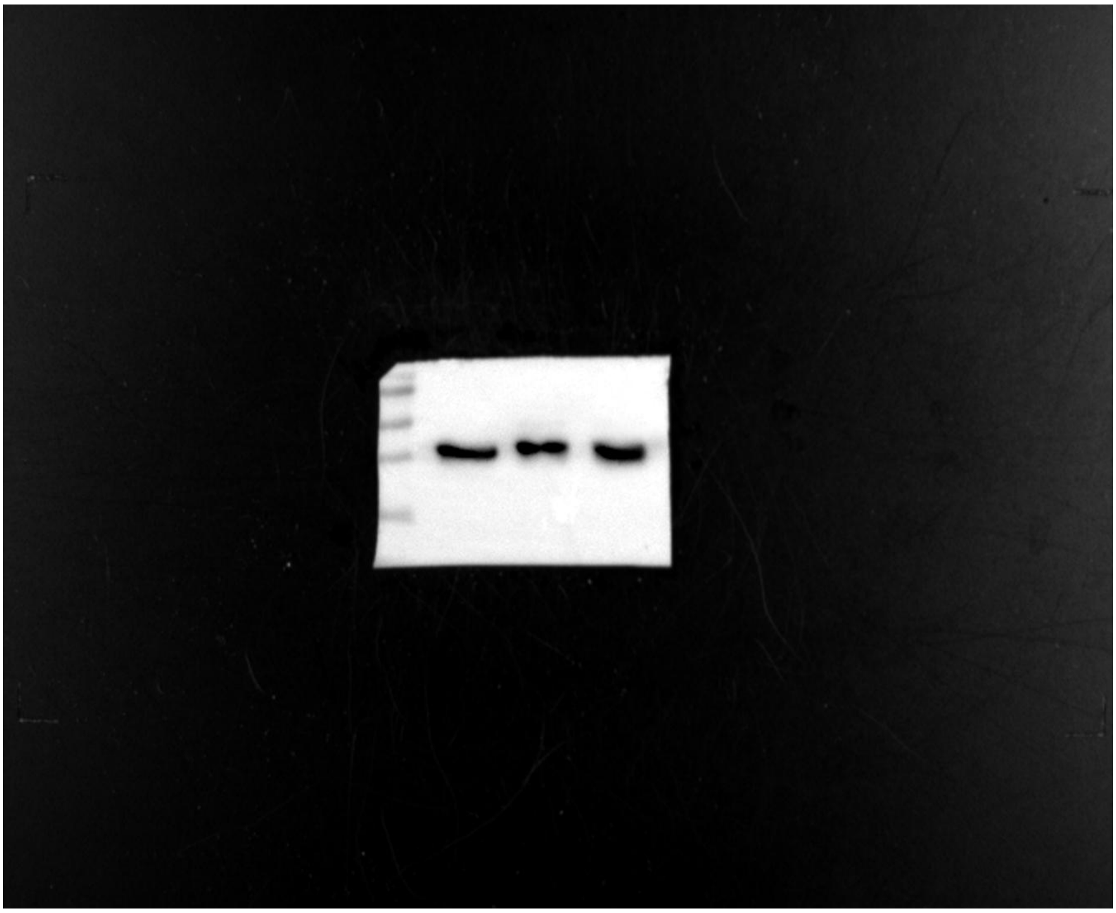

fig.6 P38 MAPK-3

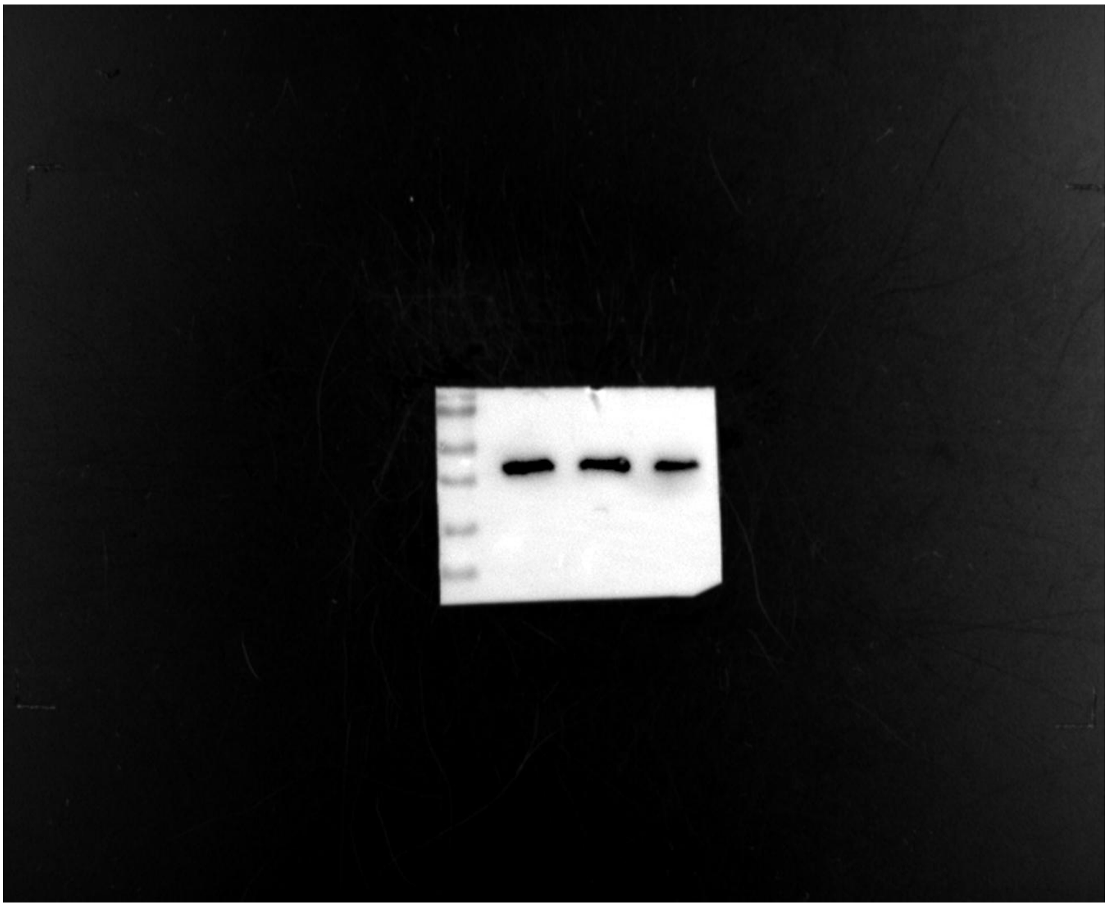

fig.6 p-P38 MAPK-1

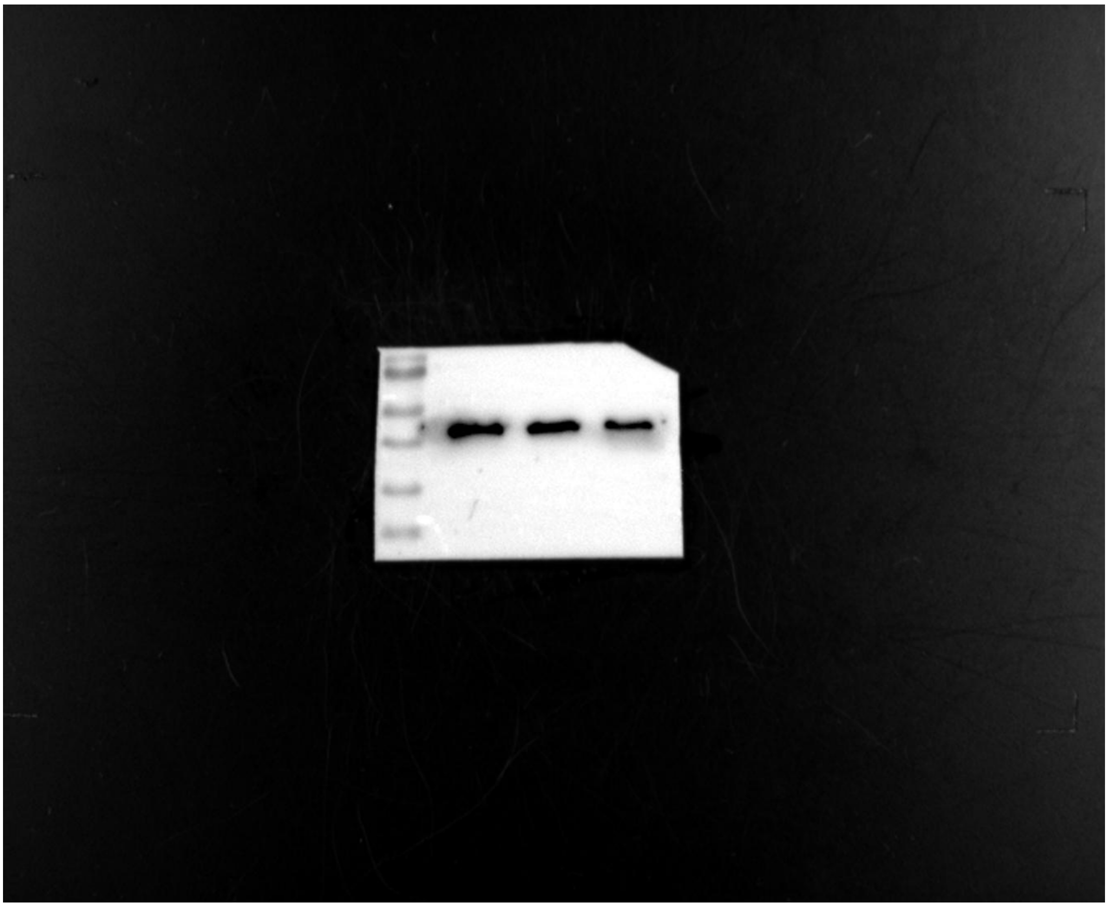

fig.6 p-P38 MAPK-2

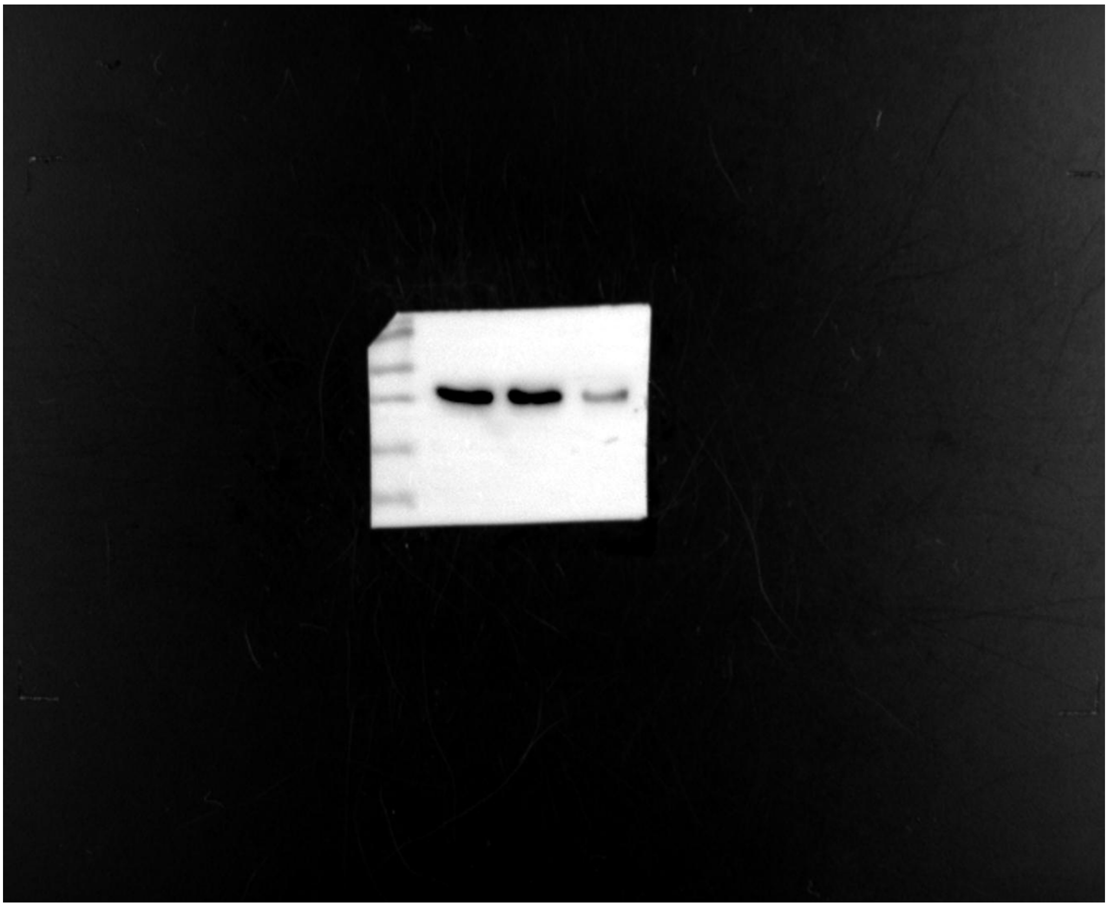

fig.6 p-P38 MAPK-3
